# Long-read single-molecule maps of the functional methylome

**DOI:** 10.1101/gr.240739.118

**Published:** 2019-04

**Authors:** Hila Sharim, Assaf Grunwald, Tslil Gabrieli, Yael Michaeli, Sapir Margalit, Dmitry Torchinsky, Rani Arielly, Gil Nifker, Matyas Juhasz, Felix Gularek, Miguel Almalvez, Brandon Dufault, Sreetama Sen Chandra, Alexander Liu, Surajit Bhattacharya, Yi-Wen Chen, Eric Vilain, Kathryn R. Wagner, Jonathan Pevsner, Jeff Reifenberger, Ernest T. Lam, Alex R. Hastie, Han Cao, Hayk Barseghyan, Elmar Weinhold, Yuval Ebenstein

**Affiliations:** 1 School of Chemistry, Center for Nanoscience and Nanotechnology, Center for Light-Matter Interaction, Raymond and Beverly Sackler Faculty of Exact Sciences, Tel Aviv University, Ramat Aviv 6997801, Israel;; 2 Institute of Organic Chemistry RWTH Aachen University, D-52056 Aachen, Germany;; 3 Center for Genetic Medicine Research, Children's National Health System, Children's Research Institute, Washington, DC 20010, USA;; 4 Kennedy Krieger Institute and Departments of Neurology and Neuroscience, The Johns Hopkins School of Medicine, Baltimore, Maryland 21205, USA;; 5 Bionano Genomics, Incorporated, San Diego, California 92121, USA

## Abstract

We report on the development of a methylation analysis workflow for optical detection of fluorescent methylation profiles along chromosomal DNA molecules. In combination with Bionano Genomics genome mapping technology, these profiles provide a hybrid genetic/epigenetic genome-wide map composed of DNA molecules spanning hundreds of kilobase pairs. The method provides kilobase pair–scale genomic methylation patterns comparable to whole-genome bisulfite sequencing (WGBS) along genes and regulatory elements. These long single-molecule reads allow for methylation variation calling and analysis of large structural aberrations such as pathogenic macrosatellite arrays not accessible to single-cell second-generation sequencing. The method is applied here to study facioscapulohumeral muscular dystrophy (FSHD), simultaneously recording the haplotype, copy number, and methylation status of the disease-associated, highly repetitive locus on Chromosome 4q.

DNA methylation of the five-carbon of cytosine is the most studied and among the most significant epigenetic modifications (Bird 2002). In mammalian DNA, methylation mostly occurs on cytosine residues within CpG dinucleotides (DNA motifs in which the cytosine is followed by a guanine residue), and 70% of such dinucleotides are methylated (Jabbari and Bernardi 2004). Approximately 60% of human gene promoters contain clusters of CpGs referred to as CpG islands (CGIs) (Esteller 2002). CpG methylation plays an essential role in the regulation of gene expression, with the general notion that hypermethylation of promoters suppresses gene expression (Jones 2012). Thus, the methylation status of gene promoters may predict gene activity and relate DNA methylation transformations in development and disease to gene expression and protein abundance.

Another genetic feature regulated by CpG methylation is repetitive arrays. These variable copy number elements are homologous DNA sequences that are identical or highly similar (Schmid and Deininger 1975; Batzer and Deininger 2002). Many repetitive elements are mobile and can transpose across the genome, perform homologous recombination events, and promote dynamic genomic transformations (Duyao et al. 1993; Batzer and Deininger 2002; Mather et al. 2011). This unstable nature explains their size variability, both among different individuals and between different cells of the same individual (Edwards et al. 1992; van der Maarel et al. 2000; Batzer and Deininger 2002). Typically, arrays are characterized by the number of units comprising them, which has been shown to affect their activity (Chamberlain et al. 1994). Methylation of repeat units adds another dimension of variability to these elements; locally it may regulate the function of individual units, and globally it can change the effective number of units in an array, altering its activity. In this context, methylation levels of repetitive DNA have been shown to be correlated with repeat-related genetic diseases (Balog et al. 2012; Pook 2012), as well as various types of cancer and their severity (Hansen et al. 2011). One example of a repeat-related disease, addressed in this work, is facioscapulohumeral muscular dystrophy (FSHD), one of the most common forms of muscular dystrophy, affecting approximately one in 7000–20,000 individuals (INSERM and French Ministry of Health 2008; Sacconi et al. 2013; Gaillard et al. 2014; Huichalaf et al. 2014).

The gold-standard method for studying CpG methylation is bisulfite sequencing, often used for whole-genome, base-pair resolution methylation profiling by second-generation sequencing (whole-genome bisulfite sequencing [WGBS]) (Cokus et al. 2008; Lister et al. 2008). As such, it provides an averaged representation of a population's methylation state at each cytosine residue (Korshunova et al. 2008). The main shortcomings of WGBS are that high-coverage sequencing (over 20× coverage) requires multiple sequencing lanes, which can be costly, and the possible need to sequence an untreated genomic DNA sample in parallel, in order to characterize individual-specific genomic loci and distinguish bisulfite conversion from native single-point mutations.

Reduced representation bisulfite sequencing (RRBS) significantly lowers sequencing costs by sampling a small subset of CpGs in order to report on the genome-wide methylation profile. Commonly, the restriction enzyme MspI is used to cut DNA at CCGG sites, followed by size selection to enrich for fragments that end within CGIs. Bisulfite conversion and sequencing result in base-pair resolution information sparsely distributed along the genome at regions dense in CCGG sites. RRBS captures ∼60% of gene promoters, thus producing crucial regulatory information while requiring very little input sample (Gu et al. 2011). The low input implies that fewer reads are necessary for accurate sequencing, allowing for high-throughput, low-cost methylation analysis for clinical and single-cell applications (Guo et al. 2013; Nagano et al. 2013). Nevertheless, bisulfite sequencing and RRBS have limited accessibility to repetitive regions owing to the inherent limitations of short-read sequencing and are often unable to quantify the length and arrangements of the repeats (Treangen and Salzberg 2011). Thus, in many cases, the genetic and epigenetic characteristics of repetitive elements are still unknown. With this in mind, it is possible that some of the many second-generation sequencing-inferred sequence duplications are in fact longer repetitive elements. Moreover, the variability of repetitive elements and methylation patterns is often masked in the averaged second-generation sequencing results, as both tend to display somatic mosaicism, manifesting different structures in different cells (van der Maarel et al. 2000).

Recent advances in third-generation sequencing methods, such as Pacific Bioscience's single-molecule real-time (SMRT) sequencing, and Oxford Nanopore Technologies’ nanopore sequencing, enable base-pair resolution sequencing of long, single DNA molecules. Sequencing reads may span tens of kilobase pairs, appropriate for characterization of medium-scale SVs and repeats (Yang et al. 2015; Morioka et al. 2016; Suzuki et al. 2016; Rand et al. 2017; Simpson et al. 2017). However, third-generation sequencing methods suffer from relatively low throughput. Consequently, although these methods allow long-read WGBS and present the potential to detect DNA modifications directly, it is costly to do so with high coverage.

DNA optical mapping (Meng et al. 1995; Michaeli and Ebenstein 2012; Levy-Sakin and Ebenstein 2013; Levy-Sakin et al. 2014; Nilsson et al. 2014; Vranken et al. 2014) stands out as an attractive approach for studying large genomic rearrangements such as repeat arrays. This method consists of a set of techniques for stretching chromosomal DNA molecules up to several megabase pairs in length, followed by imaging of these fragments using fluorescence microscopy. Image processing is then used to read out a fluorescently labeled barcode along each molecule. This barcode provides genetic information such as the genomic locus of the detected molecule, as well as the size and number of large repeat units. The most advanced method for optical genome mapping involves linearizing and uniformly stretching fluorescently barcoded DNA molecules in highly parallel nanochannel arrays. This technique, commercialized by Bionano Genomics, is capable of genetic mapping and automated copy number analysis on a genome-wide scale (Cao et al. 2014; Mostovoy et al. 2016; Dixon et al. 2018).

The use of standard fluorescence microscopy provides a mapping resolution of ∼1 kbp, which can be enhanced to 150 bp using super-resolution techniques (Jeffet et al. 2016). However, fluorescence presents the potential of obtaining several types of information simultaneously from the same DNA molecule by using different colors. Labeling different genomic features allows studying epigenetic marks and DNA damage lesions in their native genomic context on the single-molecule level (Michaeli et al. 2013; Zirkin et al. 2014; Nifker et al. 2015). To facilitate such multiplexing, we have developed an enzymatic labeling reaction that can distinguish methylated from nonmethylated cytosines. We have previously shown that this reaction can be used to detect and quantify methylation levels in synthetic DNA molecules translocated through solid-state nanopores (Gilboa et al. 2016). By combining this labeling method with Bionano Genomics genome mapping technology, we create a hybrid genetic/epigenetic fluorescent barcode for every DNA molecule. The detected molecular barcodes allow assigning the methylation profiles to their specific genomic locations, thus enabling the study of DNA methylation patterns over large genomic fragments at single-molecule resolution.

Here we aim to harness the reduced representation concept to report on the methylation profiles of long individual chromosome segments by optical genome mapping. Reduced representation optical methylation mapping (ROM) simultaneously captures large-scale structural and copy number variants together with their associated methylation status.

## Results

### ROM provides genome-wide methylation profiles of genes and regulatory elements

**Figure 1.**
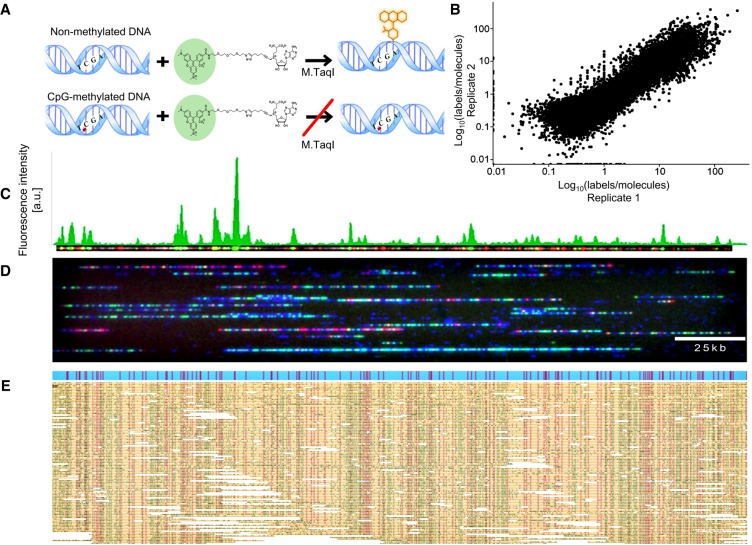
ROM experimental scheme. (*A*, *top*) M.TaqI catalyzes the transfer of a TAMRA fluorophore from the cofactor AdoYnTAMRA onto the adenine residue that lies within its TCGA recognition site. (*Bottom*) If the CpG nested within the M.TaqI recognition site is methylated, the reaction is blocked. (*B*) Scatter plot comparing nonmethylation levels in two biological ROM replicates in 10-kbp windows along the human genome. (*C*) Representative dually labeled molecule (red dots indicate genetic barcode; green dots, methylation profile). The molecule's ROM fluorescence intensity profile is presented in green *above* the molecule image. (*D*) Representative field of view of DNA molecules (blue) fluorescently labeled in two colors and stretched in nanochannel arrays. Red dots indicate genetic labels; green dots, methylation profile. (*E*) Digitized representation of single molecules (yellow) aligned to an in silico generated reference (blue) according to their distinct genetic barcode (red dots). The positions of epigenetic labels (green dots) are inferred from alignment results.

The bacterial methyltransferase M.TaqI methylates the adenine at TCGA sites. This enzyme can be “tricked” into incorporating a fluorophore instead of a methyl group by using a synthetic cofactor analog (Dalhoff et al. 2006; Klimašauskas and Weinhold 2007; Hanz et al. 2014). However, the M.TaqI reaction is blocked when the nested CpG dinucleotide in the TCGA recognition site is methylated or hydroxymethylated (Fig. 1A; Supplemental Fig. S1; McClelland and Nelson 1992). Consequently, this labeling reaction acts as a fluorescent reporter for nonmethylated CpGs within TCGA sites. When combined with existing optical genome mapping technology as a second fluorescent color, methylation profiles of individual DNA molecules can be inferred from the genomic locations of the detected labels.

We applied ROM to DNA extracted from a lymphocyte cell line and primary human whole blood. Genomic DNA was first nick-labeled to create a distinct pattern of genetic labels along the DNA (Das et al. 2010). Next, a second layer of information was added by labeling only nonmethylated CpGs with a second fluorophore. The labeled DNA was then loaded into nanochannel array chips for dual-color optical genome mapping on a Saphyr instrument (Bionano Genomics) (Fig. 1C,D). The global amount of epigenetic labels, which is quantified automatically, enables the comparison of relative global methylation levels in different samples (Supplemental Fig. S2). For genome mapping, single DNA molecules exceeding 150 kbp in length were aligned to the human genome, and alignment results were used to generate ROM profiles displaying levels of nonmethylation in CpG sites along the genome with a resolution of 1 kbp. A typical experiment will currently cost approximately $1000 and yield 50× to 100× genome coverage with highly consistent population-level methylation patterns between replicates (Fig. 1B,E).

To validate the results produced by ROM, we first wanted to estimate the efficiency of the M.TaqI labeling reaction. Protection assays using the restriction enzyme R.TaqI (Supplemental Fig. S3) confirmed that >90% of nonmethylated CpGs are labeled. Moreover, labeling performed on DNA that was methylated beforehand using the CpG MTase M.SssI resulted in a labeling efficiency of ∼0.08%, confirming the specificity of the reaction (Supplemental Fig. S3). Additionally, ROM profiles produced for two biological replicates of the GM12878 cell line were correlated on a genome-wide scale (Pearson correlation coefficient 0.74 in 10-kb windows) (Fig. 1B; Supplemental Fig. S4; Supplemental Table S1). Although sequencing replicates tend to show higher correlation, our results provide further proof for the reproducibility of the method.

**Figure 2.**
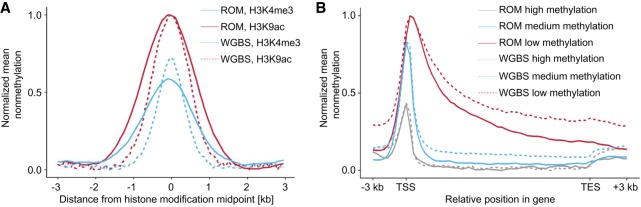
Global comparison of ROM and WGBS results in functional genomic elements. (*A*) Nonmethylation levels as a function of distance from histone modification peaks, for ROM and WGBS (blue indicates H3K4me3; red, H3K4ac). (*B*) Nonmethylation levels across gene bodies, in correlation with overall gene methylation level, determined by WGBS (red indicates low methylation level; blue, medium methylation level; gray, high methylation level). Gene lengths were normalized to 15 kbp, and 3 kbp was added upstream of the TSS and downstream from the TES.

We then turned to validate the quality of ROM profiles by comparing our results from whole blood to available WGBS data. To this end, we first compared the distributions of nonmethylated CpGs along gene bodies and regulatory histone modifications (Fig. 2). Although M.TaqI only has access to 5.5% of all CpGs, the probed TCGA sites represent approximately half of gene promoters and can hence provide valuable regulatory information. For histone modifications, it is clear from this meta-analysis that the ROM profiles are similar to those generated by WGBS, both in shape and relative intensities. Although the ROM distributions are wider, because of the limitations of optical resolution, they correlate well with the expected methylation status of these regions (Kriukienė et al. 2013). For validating the distribution in gene bodies, we divided the genes into three subgroups, according to their overall methylation level in the WGBS results. ROM produced distributions highly similar to WGBS, reporting depleted methylation levels around the transcription start site (TSS), as expected.

**Figure 3.**
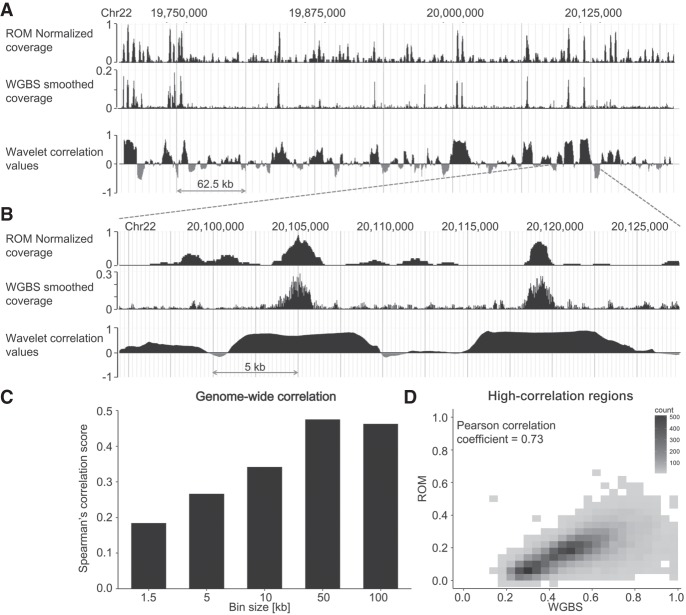
Genome-wide correlation between WGBS and ROM. (*A*) Comparison of coverage produced by both methods in a representative 500-kbp region from Chromosome 22 and the locus-specific wavelet decomposition correlation values in 10-kbp windows centered at each genomic position. (*B*) A zoom-in on a 40-kbp region from the same locus. In both cases, the WGBS data shown were inverted to represent nonmethylation levels and were smoothed using the wavelet transform. (*C*) Spearman's rank-order correlation values for genome-wide comparison of ROM and WGBS in different window sizes. (*D*) 2D density plot of nonmethylation levels in ROM in WGBS for regions identified as highly correlated by wavelet decomposition analysis.

To quantitatively assess the correlation between ROM and WGBS, we compared the methylation levels produced by both methods in varying window sizes along the genome (Fig. 3C; Supplemental Fig. S5A). Correlation was relatively low for small bin sizes (1–10 kb), probably because of the reduced-representation nature of ROM and its resolution limit, but was increased for larger windows (50–100 kb). Moreover, for larger window sizes correlation was higher in windows with higher methylation content (Supplemental Fig. S5B), a fact that can be addressed in the future by generating ROM profiles based on the fluorescence intensities of the labels and not solely on their locations.

As the correlation between ROM and WGBS is inherently limited by the correlation between TCGA content and CpG content in each genomic region, we sought to further explore which genomic loci are represented accurately by ROM. Accordingly, we created a genome-wide, locus-specific correlation track between the two data sets. To account for the difference in scaling and resolution of the two methods, this correlation track was produced by the wavelet correlation pipeline used in the ENCODE Project (The ENCODE Project Consortium 2007). We performed wavelet smoothing on the WGBS data and generated a locus-specific wavelet decomposition correlation track between WGBS and ROM (Fig. 3A,B). We then focused on regions with correlation values exceeding 0.5 (Fig. 3D), spanning 10.32% of the entire genome, and examined which functional elements are represented by these high-correlation regions. The percentages of represented elements of several genomic features are detailed in Table 1. Despite the low sampling rate of ROM, it is able to reliably characterize approximately half of some of the most important methylation regulated elements in the genome and may prove useful in studying large-scale methylation patterns in these regions.

**Table 1.**
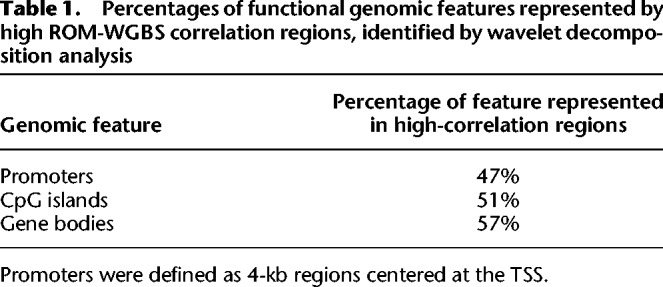
Percentages of functional genomic features represented by high ROM-WGBS correlation regions, identified by wavelet decomposition analysis

**Figure 4.**
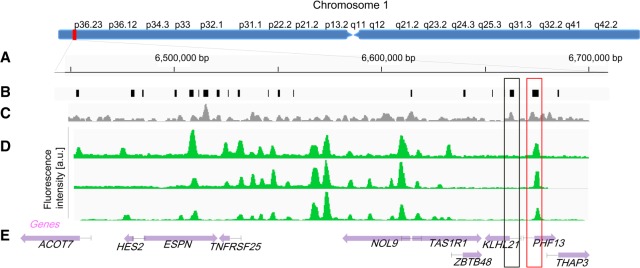
Comparison of long-range methylation profiles of single molecules. (*A*) Global view of Chromosome 1: 6,350,000–6,670,000 (bp). (*B*) Locations of CpG islands across the specified region. (*C*) Density of M.TaqI sites across the specified region in a 1.5-kb sliding window. (*D*) ROM fluorescence intensity profiles of three detected molecules aligned to the specified regions based on genetic labels (*P*-value <10^−20^). (*E*) Gene body locations and corresponding HGNC gene symbols. Each gene is displayed as a purple arrow indicating gene orientation. (*B*–*E*) Black and red rectangles indicate methylated and nonmethylated gene promoters overlapping with CGIs, respectively.

One of the main advantages of ROM is the ability to directly compare between single-molecule long-range methylation patterns spanning hundreds of kilobase pairs, made possible by the extremely long reads generated using this method. Such a comparison is shown in Figure 4, which depicts ROM profiles from genomes of three primary white blood cells aligned to the same 250-kbp region on Chromosome arm 1p. The presented methylation profiles are highly similar, and the locations of some of the fluorescent peaks correlate with locations of CGIs, indicating that these islands are nonmethylated. The small variations in pattern between the different molecules can be attributed to common variations in methylation status among white blood cells (Reinius et al. 2012). Furthermore, by using these long maps, it is possible to examine the methylation pattern of several genes, in relation to each other, for each individual genome. This information is inaccessible with second-generation sequencing because of the short available read lengths. For instance, two CGIs associated with the promoters of the genes *PHF13* and *KLHL21* are marked in Figure 4 by red and gray boxes, respectively. Although the promoter of *PHF13* was found to be nonmethylated in all molecules, the CGI residing in the promoter of *KLHL21* was detected as fully methylated. These findings are in agreement with WGBS results. However, although in the WGBS data long-range interactions are lost for individual cells, ROM methylation patterns are reported individually for each molecule, in a single-cell-like manner, and the methylation status for several more distant genes, promoters, and enhancers is also available.

### Simultaneous quantification of copy number and methylation state in DNA tandem repeats

Macrosatellite arrays, repetitive DNA that spans up to millions of base pairs across the genome, are extremely challenging for analysis by second-generation sequencing. Analysis is further complicated by the recent understanding that DNA methylation plays a crucial role in the function of these regions. One example of the significance of methylation in such regions is the D4Z4 array on Chromosome 4, which is directly related to the muscular dystrophy FSHD (Cabianca and Gabellini 2010). Recent evidence shows that both the number of D4Z4 repeats and their methylation status constitute the genotype of the disease, dictating whether the individual manifests disease symptoms or not. Commonly, healthy individuals carry an array of more than 10 repeats. In FSHD1, there is a reduction in D4Z4 repeats and hypomethylation of the region. However, even long arrays result in FSHD symptoms in the presence of hypomethylation by mutation in the *SMCHD1* gene (a condition termed FSHD2), whereas FSHD1 carriers of short but highly methylated repeat arrays may not manifest disease symptoms (Gaillard et al. 2014; Huichalaf et al. 2014). The multiple combinations of copy number and methylation levels result in a broad range of possible variations that are correlated with disease severity and manifestation (Sacconi et al. 2013; Gaillard et al. 2014).

**Figure 5.**
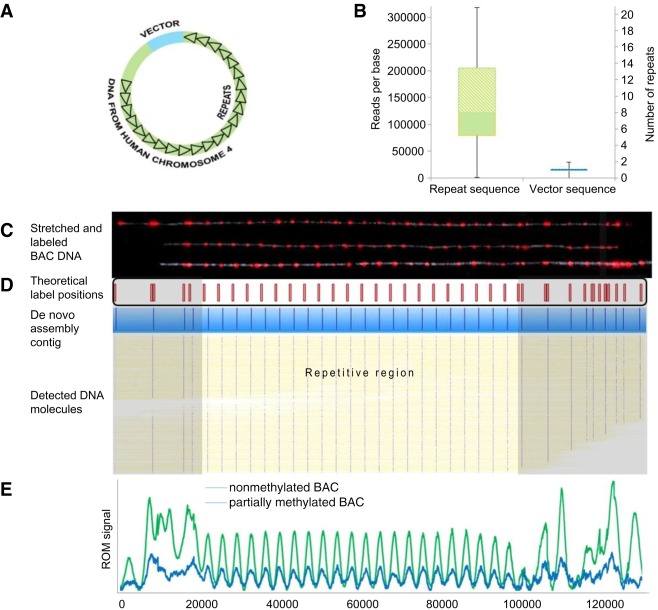
Copy number analysis. (*A*) The FSHD BAC model system contained a D4Z4 repeat array of unknown size (black triangles), genomic DNA upstream of the repeat array of unknown length (green), and the cloning vector (blue). (*B*) Box plots displaying the 25th, median, and 75th percentile (*bottom*, *middle*, and *top* of the box) of read coverage values along the repeat region (*left* box) and the nonrepetitive region (including the vector and the nonrepetitive genomic DNA; *right* box). The scale on the *right* is normalized to the median coverage along the nonrepetitive region. (*C*) Representative images of three intact model molecules labeled with Nb.BsmI (red dots) and stretched on modified glass surfaces. The labeling pattern can be aligned to the reference map presented *below* the images (expected labeling locations are shown in red). (*D*) Six hundred twenty-seven digital representations of labeled DNA molecules (yellow, blue dots represent detected labels) stretched and imaged in a nanochannel array chip. Consensus map of de novo assembly of the molecules is displayed in blue. The nonrepetitive regions are highlighted in gray (*E*) Comparative ROM profiles of nonmethylated and partially methylated BAC samples. Normalized averaged profiles of detected ROM labels are presented for the nonmethylated sample (green; 18,074 molecules) and partially methylated sample (blue; 9089 molecules). Each peak in the repetitive region corresponds to one repeat unit.

To show the capabilities of optical mapping for simultaneous copy number quantification and DNA methylation detection, we studied a model system of FSHD-associated D4Z4 repeats from a healthy human individual cloned into the CH16-291A23 bacterial artificial chromosome (BAC) (Fig. 5A). We first attempted to evaluate the copy number of the studied array by second-generation sequencing read-depth analysis (Yoon et al. 2009). We reasoned that the ratio between the number of reads representing the repeat unit and the number of reads detected for a single copy region would provide the array copy number. To assess the D4Z4 copy number, purified BAC DNA was sequenced to a read depth of 15,000×, and all sequencing reads were aligned to a reference of the BAC containing only one repeat. The average variation in read coverage along the nonrepetitive sequence was 25% of the mean read depth, whereas read coverage along the repeat sequence was much more variable, with an average variation of 63% (Supplemental Fig. S6). The estimated number of D4Z4 repeats along the BAC was calculated as eight, based on the ratio between the median read coverage values along the repetitive and nonrepetitive sequences (Fig. 5B). However, the large standard deviation values show the unreliability of this method, as well as the sensitivity of PCR amplification and second-generation sequencing to the exact content of the investigated sequence.

We next turned to make use of the advantages of optical mapping to estimate the copy number by directly visualizing the repeats along stretched DNA molecules. We created a distinct fluorescent pattern for the repeat array by using an enzyme that has a single recognition site in the 3.3-kbp-long repeat sequence (Nb.BsmI or Nb.BssSI). The labeled DNA was stretched and immobilized for visualization on modified glass slides (Fig. 5C), using a simple microfluidic scheme (see Supplemental Material). This method allowed fluorescence imaging of the entire DNA contour and localization of individual fluorescent labels along the DNA (Supplemental Fig. S7). The repetitive region can be distinguished by the equally spaced labels, each representing a repeat, and copy number was quantified by counting the labels, resulting in 22 repeats. The same sample was loaded onto an Irys instrument (Bionano Genomics), which facilitates high-throughput DNA stretching and imaging in nanochannel array chips. The post-imaging analysis, performed by the IrysView software suite, involved automatic label detection and de novo assembly of the molecules into a contiguous consensus barcode. The resulting consensus map was created in an unsupervised manner based on label patterns from approximately 1000 detected molecules (Anantharaman and Mishra 2001; Pendleton et al. 2015). When comparing the nonrepetitive region of this consensus map to the one predicted from the known sequence, an almost perfect match was obtained (*P*-value <10^−43^) (Fig. 5D). The repetitive region of the consensus map displays 22 labels; however, as the first or last repeat may be truncated and unlabeled (Dini and Lipsick 1993; Satović et al. 2016), these 22 labels indicate 22 ± 1 D4Z4 repeats. The exact size of the array can be validated by measuring the length of the array directly from the consensus map. These results show the potential of the technique for genetic diagnosis of FSHD.

For methylation analysis, we performed ROM as an overlay on the repetitive genetic barcode. We used red fluorophores for the genetic barcode and green fluorophores for methylation mapping. Supplemental Figure S7A shows the unique pattern created by M.TaqI along the nonmethylated BAC (Jeffet et al. 2016), highlighting nonmethylated repeat units. To simulate the native state of DNA, in which repeat arrays are methylated to variable degrees, we partially methylated the DNA using the CpG-specific DNA MTase M.SssI. We repeated the dual-labeling reaction on the partially methylated sample, as well as on a nonmethylated control, and analyzed both on the nanochannel array chips. After image analysis, we used the red genetic labels for automated de novo assembly and generated the consensus map with 22 repeats as described earlier. With thousands of molecules now aligned to the consensus map, we could compare the methylation patterns generated by ROM on the nonmethylated and partially methylated samples. Figure 5E shows averaged methylation profiles generated from the molecules detected in each data set. The detected number of labels at the expected positions was high in the nonmethylated sample, whereas the partially methylated DNA sample displayed significantly lower labeling. It is clear from this plot that the methylated CpGs are distributed uniformly among the repeat units, in line with the fact that the partial methylation was random and uniform for all repeats in the array. These results show that ROM provides not only single-molecule and single-repeat resolution but also an assessment of the average methylation status for each repeat in the array across a population of different DNA molecules.

Because 1000× coverage is not feasible for a human whole-genome experiment at a reasonable cost, we wanted to estimate the minimal coverage needed for detection of a contracted array unit. To reliably report on the number of repeat units, at least one molecule spanning the complete array needs to be sampled. We estimated that a contracted array, containing at most 10 repeat units, would span ∼50 kb, including the flanking regions needed for unambiguous alignment. We therefore divided the genome into nonoverlapping 50-kb windows and calculated the percentage of such regions completely covered by at least one molecule in a typical experiment (Supplemental Fig. S8; Supplemental Table S2). Random subsampling of the molecules showed that ∼96% of target regions are covered by at least one molecule at 20× coverage, much lower than the coverage obtained in a single experiment.

### ROM enables genetic/epigenetic diagnosis of FSHD

**Figure 6.**
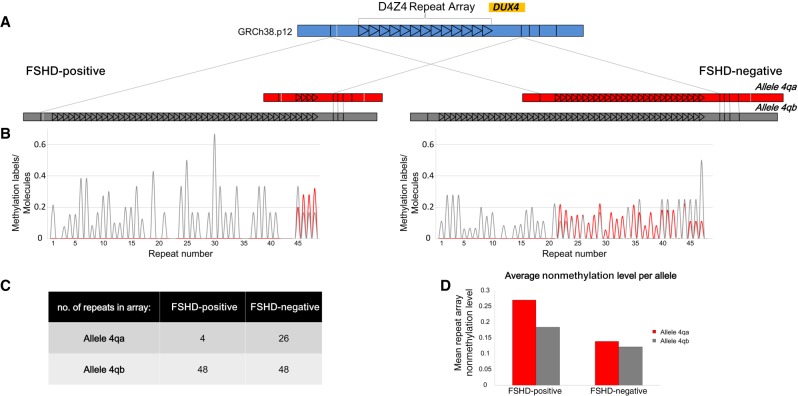
Copy number and methylation analysis of the pathogenic contraction on Chromosome arm 4q of an FSHD patient compared with a healthy individual. (*A*) De novo assembly consensus optical maps of the two alleles, 4qA (red) and 4qB (gray) for both samples. Alignment to the in silico–generated map of this region (blue) is indicated by gray lines. Each repeat is represented by a black triangle. (*B*) Average profiles of detected ROM methylation labels for the two individuals (red indicates 4qA; gray, 4qB). Each peak in the plot corresponds to the average nonmethylation level of one repeat unit. (*C*) Table summarizing the number of detected repeat units in each allele. (*D*) Bar plot displaying the mean ROM nonmethylation value in the complete repeat array in both alleles for both samples.

Finally, to show the diagnostic potential of the method, we performed ROM on DNA extracted from myocytes of a donor previously diagnosed with FSHD, as well as from those of the donor's unaffected sibling. A comparison between the detected copy number of the D4Z4 repeats and the methylation status of each repeat is depicted in Figure 6. De novo assembly of the region based on genetic labels detected on long, single molecules allowed us to distinguish between the two very similar 4qA and 4qB alleles, each with a distinct copy number of D4Z4 repeats (Fig. 6A). In the healthy donor sample, both alleles contained more than 10 repeats (Fig. 6C), whereas the pathogenic 4qA allele in the patient's sample contained only four repeat units. Moreover, averaged ROM methylation profiles produced for the two samples (Fig. 6B) indicated that the patient's mean methylation level per repeat was lower than the healthy individual's, in both alleles (Fig. 6D). This trend is in line with expected hypomethylation of this repeat array in FSHD patients. Additionally, we performed ROM on DNA extracted from whole blood of another patient tested by traditional clinical genetic assessments based on pulsed field electrophoresis. The number of identified repeats was in line with the genetic test while providing the additional methylation profile (Supplemental Fig. S9). These results show the complementarity of genetic and DNA methylation information generated by ROM and show the utility of this optical mapping approach for characterizing the genetic and epigenetic profile of FSHD, as well as other macrosatellite arrays and large structural variants.

## Discussion

Optical genome mapping has recently been used to map whole-human genomes at high coverage and to highlight genetic variability between individuals with great detail (Cao et al. 2014; Mostovoy et al. 2016). This work adds an epigenetic component to optical maps, simultaneously providing genetic and methylation profiles for individual DNA molecules spanning hundreds of thousands of base pairs. By using optical genome mapping technology, combined with DNA MTase-assisted methylation detection, we create a ROM, which reports on the methylation status of ∼50% of gene promoters (Table 1; Supplemental Fig. S10; Supplemental Table S3). Optical mapping allows genomic information to be read directly from individual, unamplified, long fragments of DNA, thus mitigating the bias of large structural and copy number variation in single-cell sequencing experiments and eliminating the need for bisulfite conversion. Unlike RRBS, ROM is not limited to CpG-rich regions and provides a reduced representation methylation status of the entire genome. By automatic quantification of the amount of nonmethylated CpGs, ROM enables high-throughput analysis of global methylation levels across multiple cell types. Additionally, ROM provides long-read methylation profiles of single DNA molecules. The resulting methylation patterns detected around genes and regulatory elements throughout the genome are in line with those produced by WGBS. However, the long-read property of ROM may allow studying the methylation profiles of genes together with remote regulators such as distant enhancers and *cis* elements. Furthermore, recent reports have linked several types of cancer to changes in methylation patterns of long genomic regions spanning 5 kbp to 10 Mbp, termed partially methylated domains (PMDs) (Madakashira and Sadler 2017). The methylation status of lamina-associated domains (LADs), which correlates with changes in nuclear organization, has also been associated with tumorigenesis (Timp and Feinberg 2013). It therefore may be of importance to be able to examine the overall methylation status of large blocks of DNA in situations in which changes in methylation patterns become sporadic, with high variability between cells. ROM may enable the characterization of the methylation profiles of these long regions in a single-cell-like manner, providing insight into epigenetic changes associated with cancer progression.

Another area in which ROM has a particular advantage over existing technologies is the study of DNA methylation of large structural variants and repetitive arrays. DNA repeats are dynamic regions, showing high variability both in length and methylation status, and thus may differ significantly between individuals (Edwards et al. 1992; Lemmers et al. 2012). It is becoming increasingly accepted that the full profile of genomic structural variation, including DNA repeats, is directly linked to health and susceptibility to disease (Duyao et al. 1993; Choi et al. 2009). ROM offers single-molecule level information on the size of the region, the number of repeat units, and the methylation status of individual repeats. This detailed information is inaccessible via current genome technologies such as second-generation sequencing, DNA arrays, and quantitative PCR, which mostly provide averaged or inferred data and cannot accurately address individual repeat units.

We show the utility of ROM for characterizing the D4Z4 repeat array, in which both the size and the methylation status of the repeats affect FSHD disease manifestation (Gaillard et al. 2014). Explicitly, the reported approach can be useful for distinguishing between healthy and FSHD individuals by combining copy number and methylation level information. Moreover, the detailed view of the methylation status of individual repeats may offer new insights into the mechanism of disease and may lead to a more individualized prognosis than is being provided by current commercial testing.

The utility of ROM for characterizing methylation status in parallel with copy number may be extended to other genetic disorders. One such example is Fragile X syndrome (FXS), which has been linked to an expansion of the trinucleotide CGG repeat in the 5′ untranslated region (UTR) of the *FMR1* gene located on the long arm of Chromosome X. When such an expansion occurs, the 5′ UTR and promoter of the gene become hypermethylated (Jin 2000; Alisch et al. 2013). ROM may assist in characterizing the methylation state of this region and the long-range interactions influencing it, potentially shedding light on the mechanism of *FMR1* silencing.

Notably, the labeling concept is not limited to reduced representation, and CpG labeling enzymes such as M.SssI (Kriukienė et al. 2013) may be used to address all methylation sites. Further development of this technique may serve to map differentially methylated single-molecule patterns on a genome-wide scale, potentially allowing simultaneous genetic/DNA methylation haplotyping, as well as ultrasensitive detection of epigenetic transformations.

## Methods

### Human subjects

This study was approved by The Johns Hopkins School of Medicine institutional review board. The donor with a clinical diagnosis of FSHD1 was confirmed by the University of Iowa Diagnostic Laboratories to have a contracted D4Z4 array on a 4qA allele by pulsed-field gel electrophoresis and Southern blotting. The EcoRI/BlnI allele of 15 kbp on Chromosome 4qA corresponds to approximately four repeats, and the 27-kbp allele on Chromosome 4qB corresponds to approximately seven repeats.

### DNA samples

λ-Bacteriophage DNA (New England BioLabs [NEB]) was used as provided. BAC DNA was purified from *Escherichia coli* cells containing the CH16-291A23 BAC. Cells were cultured overnight in LB containing 12.5 µg/mL chloramphenicol (Sigma-Aldrich) at 30°C. BAC DNA was purified from the cells using the NucleoBond Xtra BAC kit (Macherey-Nagel). DNA from a NA12878 lymphocyte cell line (Coriell Institute for Medical Research), primary human blood cells, and human myocytes were purified in agarose plugs to protect DNA from shearing and maintain long DNA fragments, following Bionano Genomics’ sample preparation protocols, according to the manufacturer's instructions.

### M.TaqI-assisted labeling for ROM

To generate methylation sensitive labeling profiles we used the DNA MTase M.TaqI, which catalyzes the transfer of a carboxytetramethylrhodamine (TAMRA) fluorophore from the synthetic cofactor AdoYnTAMRA onto the adenine residue within its recognition sequence (TCGA) (Grunwald et al. 2015). The labeling reaction was performed as follows: 1 µg of DNA was reacted with 37.5 ng of M.TaqI and 40 µM of AdoYnTAMRA in labeling buffer (20 mM Tris-HOAc, 10 mM MgCl_2_, 50 mM KOAc, 1 mM DTT at pH 7.9), in the presence of 0.01% Triton X-100 and 0.1 mg/mL BSA, in a total reaction volume of 25 µL for 1 h at 60°C . The labeled DNA was reacted with 40 µg of Proteinase K (Sigma-Aldrich) for 1 h at 45°C to disassemble protein–DNA aggregates. For methylated samples, CpGs were methylated before labeling using the CpG-specific DNA MTase M.SssI (Thermo Fisher Scientific) according to the manufacturer's instructions but with twice the suggested amount of enzyme to ensure complete methylation. To obtain partial methylation, the reaction was performed using the recommended amount of enzyme, but for 75% of the recommended incubation time. Methylation was verified by digestion with the methylation-sensitive restriction enzyme HpaII (NEB) followed by gel electrophoresis to ensure that the DNA was fully or partially protected from restriction (Supplemental Fig. S3).

### Nick-labeling for optical genome mapping

DNA was prepared in a nick-labeling-repair reaction (NLR), which involves (1) either the nicking enzyme Nb.BsmI or the nicking enzyme Nt.BspQI, which generate single-strand nicks at specific recognition sites (GAATGC or GCTCTTCN, respectively); (2) a DNA polymerase enzyme, which incorporates fluorescent nucleotides at the nicked sites; and, finally, (3) a DNA ligase enzyme, which repairs the remaining single-strand breaks. For the NLR reaction involving Nt.BspQI, DNA was labeled using the IrysPrep kit (Bionano Genomics) according to the manufacturer's instructions. For Nb.BsmI-based NLR, DNA (900 ng) was first reacted with 30 units of the enzyme (NEB) in 30 µL NEBuffer 3.1 for 120 min at 65°C. Next, the DNA was reacted with 15 units of Taq DNA polymerase (NEB) in the presence of the following nucleotides: dGTP, dCTP, dATP (Sigma-Aldrich), and the fluorescent nucleotide dUTP-Atto647 (Jena Bioscience) at a final concentration of 600 nM each. The reaction was performed in a reaction buffer (ThermoPol buffer, NEB) in a total volume of 45 µL for 60 min at 72°C. Finally, the DNA was reacted with 120 units of Taq DNA ligase (NEB) with 0.5 mM NAD^+^ (NEB), in a reaction buffer (ThermoPol buffer, NEB) including 10 µM dNTP mix, in a total reaction volume of 60 µL for 30 min at 45°C. For ROM experiments, DNA was initially labeled by NLR, and then 0.05–0.5 µg of the labeled DNA was reacted with M.TaqI as described above (all reaction components were scaled down accordingly). Before M.TaqI labeling, the Nb.BsmI NLR reaction products were re-embedded in 1% agarose plugs to allow for washing in water. For washing, plugs were incubated for 10 min in water, and this step was repeated three times by replacing the water between washes. In the case of Nt.BspQI-NLR, the M.TaqI reaction was performed in the NLR buffer with the addition of 1× buffer 4 and 1× BSA (NEB), and the pH was adjusted to 8.3 using 0.1 M NaOH. Before imaging, the agarose matrix was digested using a GELase enzyme (Epicenter).

### Sample preparation, DNA stretching, and imaging

Post-labeling, BAC and λ DNA were cleaned by ethanol precipitation as has been described previously (Grunwald et al. 2015). Genomic DNA was cleaned by embedding it into agarose plugs and washing these in TE buffer (see Supplemental Material). Before imaging, the labeled DNA was stained with 0.5 µM YOYO-1 (Invitrogen) for visualization of its contour; 200 mM DTT (Sigma-Aldrich) was added to the reaction to prevent photobleaching and DNA breaks. To stretch the DNA from its random coil conformation into a linear form, allowing imaging of its contour, we used two types of experimental schemes: The first stretching scheme was based on modified glass surfaces. In this approach, DNA sample solutions were flowed on glass surfaces that were chemically modified to facilitate DNA anchoring and stretching on the surface by applying capillary forces or using microfluidics (see Supplemental Material). After stretching, DNA was imaged using an epifluorescence microscope (FEI Munich) equipped with a high-resolution EMCCD IXon888 camera (Andor Technology). A 150-W xenon lamp (FEI Munich) was used for excitation with filter sets of 485/20ex and 525/30em, 560/25ex and 607/36em, and 650/13ex and 684/24em (Semrock) for the YOYO-1, TAMRA, and Atto-647 channels, respectively. For high-throughput mapping experiments, samples were analyzed in nanochannels array chips (Bionano Genomics). On the chip, DNA is forced into 45-nm-square nanochannels using an electric field and is stretched along the channel axis for imaging. This process is performed in automated cycles by the Irys instrument (Bionano Genomics) (Das et al. 2010).

### Dual-color NA12878 labeling and data generation

DNA from NA12878 was isolated using prep blood and cell culture DNA isolation kit (Bionano Genomics 80004) in two independent DNA isolation preps. DNA was labeled by NLRS DNA labeling kit (80001) using Nt.BspQI (NEB r0644) as directed, using 1200 ng of DNA nicked in a 30 µL reaction instead of the normal 40 µL reaction. After labeling (red cofactor) and ligation, 500 ng of the reaction product was labeled with M.TaqI and a custom green cofactor (Bionano Genomics), similar to the TAMRA cofactor above. DNA was incubated in 1× NEB buffer 4 + BSA, 1× NEB BSA, 60 µM M.Taq green cofactor, and 10 units of M.TaqI (NEB) for 5 h at 65°C. After the reaction, 1 µL of Puregene Proteinase K (Qiagen) was added and incubated for 60 min at 37°C. To remove excess cofactor, dialysis against 20 mL of 1× TE (pH 8.0) with a 0.1-μM dialysis membrane was performed for a total of 2 h and 45 min, making sure to protect from light. During dialysis, the droplet was moved to another spot on the membrane after 30 min because the dye binds to the membrane. After 90 additional minutes, the drop was moved once again, and dialysis was continued for 45 additional minutes. DNA was then quantitated, and 450 ng of DNA was stained in a total volume of 90 µL according to Bionano NLRS prep kit with an addition of 25 mM Tris (pH 8) and 25 mM NaCl. The samples were loaded onto Saphyr chip (Bionano Genomics) flow cells and ∼320 Gbp of data was collected for each sample on a Saphyr system (Bionano Genomics).

### Data analysis

For analysis of the high-throughput nanochannel array data, raw images were processed, and DNA molecules were detected and digitized by custom image-processing and analysis software (Irys Extract [Arielly and Ebenstein 2018], or IrysView [Cao et al. 2014] and Bionano Access [Bionano Genomics]). Genetic labels were assigned one set of coordinates along the molecules (the genetic map), and the methylation labels were assigned another set (the ROM map). One output of the detection process is the number of labels per 100 kbp, allowing direct comparison between labeling levels of different samples and allowing quantitative assessment of methylation levels (Supplemental Fig. S5).

For ROM, DNA molecules were first aligned to the genome reference based on the match between the fluorescent genetic pattern along the molecule and the pattern expected from the known reference sequence. We note that because available WGBS data are aligned to build hg19, optical data were aligned to the same reference for comparison. After alignment, the methylation maps indicated the distribution of nonmethylated CpGs along the mapped genomic regions, and methylation profiles were exported in BED format for visualization in standard genome browsers such as the UCSC Genome Browser (Kent et al. 2002; https://genome.ucsc.edu/index.html).

Details on bioinformatics analysis and comparison to WGBS data are provided in the Supplemental Material.

IrysView also enabled de novo assembly of the detected molecules into a consensus barcode or contig (genome map) and its comparison with the theoretical barcode. This unsupervised assembly was used to assess the number of repeats in the FSHD BAC model system. De novo assembly for dually labeled DNA molecules derived from myocytes was performed via tools provided in Bionano Access web server version 1.2 (Bionano Genomics). Custom R scripts were developed to calculate the methylation levels in the *DUX4* region (Supplemental Code). Briefly, the scaffolds generated via Bionano Access were used to identify all of the molecules aligning to the *DUX4* region. Then, corresponding reference positions of nicking and methylation labels were calculated using the molecule-specific CMAPs and XMAPs. These positions were used to identify the number of D4Z4 repeats in each of the human haplotypes and to quantify the number of methylation-specific labels.

Images of DNA molecules stretched on modified glass surfaces were manually aligned according to the barcode created outside the repetitive region. This unique barcode enabled detection of the starting point of the repeat array, allowing counting of individual repeat units.

### Second-generation sequencing

Purified BAC DNA was sheared using Covaris AFA (Covaris). Fragments were size-separated by electrophoresis using agarose gel, allowing selective extraction of fragments within the range of 150–300 bp. Illumina sequencing libraries were prepared using NEXTflex kit (Bioo Scientific) and paired-end sequenced using MiSeq (Illumina) to a coverage of 15,000×. Sequencing reads were de novo assembled using CLC Workbench software (CLC Bio-Qiagen).

## Data access

All raw sequencing data generated in this study have been submitted to the Sequence Read Archive (SRA; https://www.ncbi.nlm.nih.gov/sra) under accession number SRR8439260. Optical mapping CMAP and XMAP files generated in this study have been submitted to the NCBI BioProject database (https://www.ncbi.nlm.nih.gov/bioproject) as Supplemental Files under accession number PRJNA514981. Custom R scripts used to count the number of repeats in the *DUX4* region are available as Supplemental Code. Documentation is available as Supplemental File 4. Other custom Python and R scripts used in this study are available both as Supplemental Code and on GitHub: https://github.com/ebensteinLab/Irys-data-analysis. Optical mapping and wavelet correlation bedGraph files are available as a UCSC Genome Browser public session: https://genome-euro.ucsc.edu/cgi-bin/hgTracks?hgS_doOtherUser=submit&hgS_otherUserName=hilasha&hgS_otherUserSessionName=methylation%20OM. The wavelet decomposition correlation track is also available as Supplemental File 1.

## Competing interest statement

Y.E., E.W., and A.G. have filed a provisional patent application related to this work.

## Supplementary Material

Supplemental Material
