# Supplementary material for: Long-read single-molecule maps of the functional methylome
Source: Genome Res. 2019 Apr;29(4):646–56. doi: 10.1101/gr.240739.118 (PMC6442387; doi:10.1101/gr.240739.118)
Supplement: Supplemental Material [file supp_29_4_646__index.html]

Long-read single-molecule maps of the functional methylome — Supplemental Material 

# Long-read single-molecule maps of the functional methylome

## Supplemental Material

- Supplemental\_Material.pdf
- Supplemental\_code\_methometeR\_Location\_Calculation\_Negative\_Strand.r
- Supplemental\_code\_methometeR\_Location\_Calculation\_Positive\_Strand.r
- Supplemental\_code\_MethylationCount\_and\_Normalization.r
- Supplemental\_code\_automated\_wavelet\_pipe.py
- Supplemental\_code\_CWT\_DOG\_calc.R
- Supplemental\_code\_MODWT\_calc.R
- Supplemental\_File\_1.zip
- Supplemental\_File\_4.docx
- Supplemental\_File\_2.pdf
- Supplemental\_File\_3.txt
