# Supplementary material for: Long-read single-molecule maps of the functional methylome
Source: Genome Res. 2019 Apr;29(4):646–56. doi: 10.1101/gr.240739.118 (PMC6442387; doi:10.1101/gr.240739.118)
Supplement: Supplemental Material [file supp_gr.240739.118_Supplemental_Material.pdf]

Supplemental Material

Long-read single-molecule maps of the functional methylome.

Hila Sharim[1], Assaf Grunwald[1], Tslil Gabrieli[1], Yael Michaeli[1], Sapir Margalit[1], Dmitry Torchinsky[1], Rani Arielly[1], Gil Nifker[1], Matyas Juhasz[2], Felix Gularek[2], Miguel Almalvez[3], Brandon Dufault[3], Sreetama Sen Chandra[3], Alexander Liu[3], Surajit Bhattacharya[3], Yi-Wen Chen[3], Eric Vilain[3], Kathryn R Wagner[4], Jonathan Pevsner[4], Jeff Reifenberger[5], Ernest T Lam[5], Alex R Hastie[5], Han Cao[5], Hayk Barseghyan[3], Elmar Weinhold[2*], Yuval Ebenstein[1*].

[1]School of Chemistry, Center for Nanoscience and Nanotechnology, Center for Light-Matter Interaction, Raymond and Beverly Sackler Faculty of Exact Sciences, Tel Aviv University, Tel Aviv, Israel, [2]Institute of Organic Chemistry RWTH Aachen University D-52056 Aachen Germany, [3]Center for Genetic Medicine Research, Children's National Health System, Children's Research Institute, Washington, DC 20010, USA, [4]Kennedy Krieger Institute and Departments of Neurology and Neuroscience, The Johns Hopkins School of Medicine, Baltimore, MD, USA, [5]Bionano Genomics, Inc., San Diego, CA, USA.

DNA methylation detection at the single-molecule level by DNA-methyltransferase assisted labeling

DNA methyltransferase enzymes (MTases) offer an orthogonal method for DNA sequence-specific labeling as they may be "tricked", using synthetic cofactor analogs, into directly incorporating a fluorophore onto their recognition site (Hanz et al. 2014; Klimasauskas and Weinhold 2007) (Figure S1A upper panel). We have previously demonstrated how the DNA MTase M.TaqI (with the TCGA recognition sequence) can generate DNA barcodes for optical mapping and bacteriophage strain typing by transferring a fluorophore onto the target adenine in its recognition site (Grunwald et al. 2015). However, when the cytosine in the nested CpG dinucleotide contained in the M.TaqI recognition site is methylated, adenine methylation by M.TaqI is blocked (McClelland and Nelson 1992).

To test whether this property of M.TaqI could also be used to label DNA in a CpG methylation-dependent manner, the labeling reaction was applied to the 48.5 kbp long λ-bacteriophage genome, containing 121 M.TaqI recognition sites. λ-DNA was labeled with the fluorescent cofactor AdoYnTAMRA, yielding a unique continuous fluorescent signal along individual genomes that were deposited and

imaged on a microscope slide (Figure S1B, right image). After methylating all CpGs on the λ-bacteriophage genomes by *in-vitro* treatment with the CpG MTase M.SssI, no labeling was detected on the deposited λ-DNA except for very rare events. We estimated one label for every 10 λ-DNA molecules which translates to a labelling efficiency of ~0.08% for methylated DNA. We note that this labelling can also be attributed to sites that were not methylated by M.SssI or to random colocalization of free fluorophores with DNA. These data confirm that the labeling reaction is blocked by existing CpG methylation with only a fraction of a percent false positives (Figure S1B, left image).

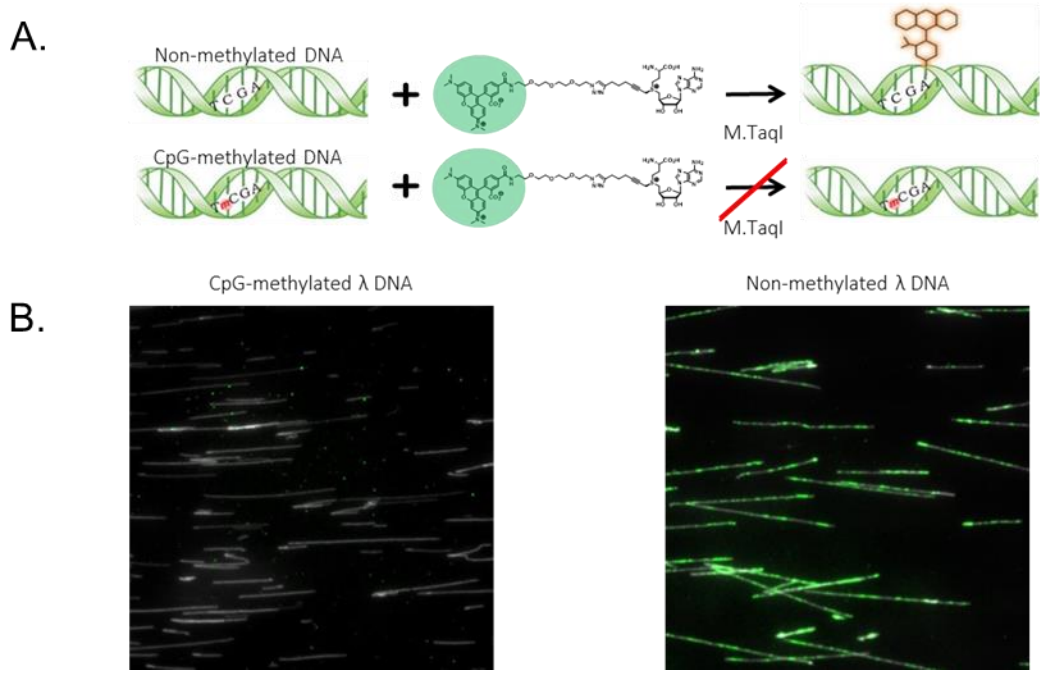

Figure S1. A. Top: M.TaqI catalyzes the transfer of a TAMRA fluorophore from the cofactor AdoYnTAMRA onto the adenine residue that lies within its TCGA recognition site. Bottom: If the CpG nested within the M.TaqI recognition site is methylated, the reaction is blocked. **B.** Non-methylated or CpG-methylated λ-DNA was reacted with M.TaqI and the fluorescent cofactor AdoYnTAMRA, and the DNA backbone was stained with YOYO-1. Labeled DNA was stretched on modified glass surfaces and imaged in two channels to visualize its contour (displayed in grey) and M.TaqI fluorescent labeling (shown in green).

Methylation-sensitive optical mapping quantifies genome-wide methylation levels

To test whether ROM can be combined with optical mapping for comparing the methylation levels of various cell types we analyzed DNA methylation levels in primary blood cells and an Epstein-Barr virus

(EBV) transformed lymphocyte cell-line, NA12878 (Coriell Institute, USA), for which reduced genome-wide methylation levels have been reported (Hansen et al. 2014).

DNA from both samples was dually labeled with genetic and methylation labels, where the genetic labels were generated using the nicking enzyme Nt.BspQI and the methylation labels were generated using M.TaqI. After labeling, the DNA was stretched in nanochannels (Bionano Genomics, Inc., San Diego, California, USA) and imaged (Figure S2A). The IrysView software suite was used to automatically detect and count the number of methylation labels along each stretched molecule. Our goal was to provide global quantification of the relative methylation levels in the two samples. In total, 4.6 Gbp were sampled for the primary blood cells DNA and 1.2 Gbp for the lymphocyte cell line. As expected (Hansen et al. 2014), EBV immortalized cell line genomes were hypomethylated compared to DNA from primary cells, with a 3.6-fold higher number of M.TaqI labels compared to the primary DNA (16.0 vs. 4.7 labels per 100 kbp; Figure S2B). These results demonstrate the utility of M.TaqI labeling for quantitative assessment of methylation levels in various samples. One immediate potential application for such an analysis is following the modulation in global DNA methylation levels during the progression of cancer and response to therapy.

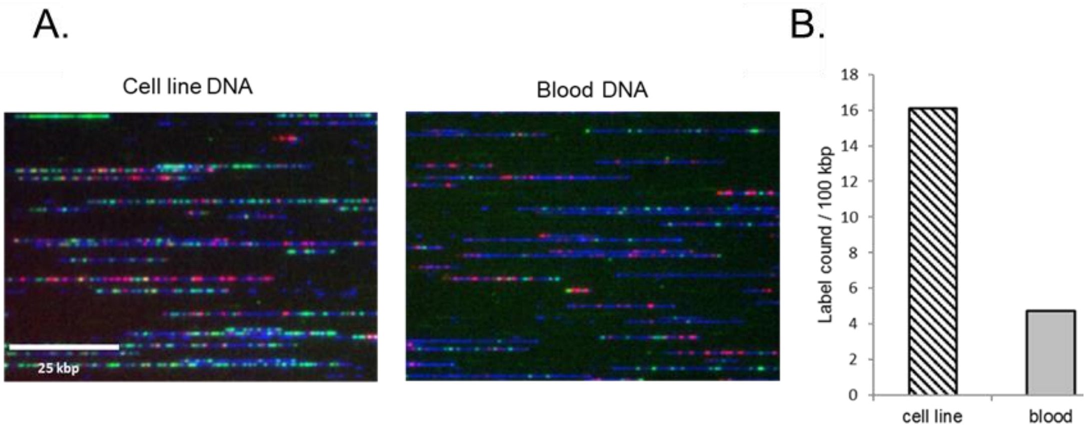

Figure S2. Methylation detection in genomic DNA. **A.** Genomic DNA from a human lymphocyte cell line culture (left image) and primary human blood cells (right image) was labeled with genetic labels (Nt.BspQI, red labels) and methylation-sensitive labels (M.TaqI, green labels), and imaged on a nanochannel array chip. Representative images from both samples are presented here. DNA backbone, stained with YOYO-1, is displayed in blue. **B.** Barplot displaying the number of detected non-methylated CpG labels per 100 kbp for both samples. Dashed bar displays the label number for the cell line DNA and gray bar for the primary blood DNA.

M.TaqI labeling efficiency and sensitivity to DNA methylation levels

We further verified the methylation sensitivity of the reaction by a bulk restriction-protection assay (Figure S3A). This type of assay is performed to test MTase activity by reacting modified DNA with the enzyme, and restriction products are analyzed using gel electrophoresis. Typically, the DNA is reacted with decreasing enzyme-to-site ratios in order to estimate the minimum stoichiometric enzyme amounts needed for complete modification. Here, non-methylated and CpG-methylated λ-DNA were compared as substrates for M.TaqI modification of the adenine in the TCGA sequence. We reacted the λ-DNA samples with both the natural cofactor AdoMet (which facilitates the enzymatic transfer of a methyl group) and the synthetic cofactor AdoYnTAMRA (which facilitates transfer of a TAMRA fluorophore) using decreasing amounts of M.TaqI. Finally, the sample was incubated with the restriction enzyme R.TaqI (New England Biolabs Inc., Ipswich, MA, USA), which has an identical TCGA recognition sequence as M.TaqI. R.TaqI is not sensitive to cytosine methylation, but cuts DNA only if the adenine in its recognition site is non-methylated or non-labeled.

Protection assays were performed as follows: Two-fold serial dilutions of DNA MTase (20 µL) were prepared by adding 2 µL M.TaqI to a solution of 38 µL reaction buffer (20 mM Tris-HOAc, 10 mM $Mg(OAc)_2$, 50 mM KOAc, 1 mM DTT, 0.01% Triton X-100, 0.1 mg/mL BSA, pH 7.9) containing λ-DNA or M.SssI-methylated λ-DNA (0.05 µg/µL, 1.56 nM, 189 nM TCGA recognition sites) and AdoMet or AdoYnTAMRA (80 µM). An aliquot of these solutions (20 µL) was removed and added to reaction buffer (20 µL) containing λ-DNA and cofactor to give a 2-fold enzyme dilution. This step was repeated several times to yield 4-, 8-, and 16-fold enzyme dilutions. The reaction mixtures were incubated at 60 °C. After 1 h, 10 units of R.TaqI restriction endonuclease (30 µL in 16.6 mM Tris-HCl, 166 mM NaCl, 8.3 mM $MgCl_2$, 0.166 mg/mL BSA, pH 8.0) were added, and the incubation continued at 60 °C for 1 h. Loading buffer was added (10 µL, 0.25% bromophenol blue, 30% glycerol), and samples (10 µL) were resolved on a 1% agarose gel in the presence of GelRed (0.1 µL stock solution/mL gel). DNA bands were visualized with a UV transilluminator (312 nm) and documented with a CCD camera equipped with a filter (540 ± 50 nm).

Digested DNA confirms the inability of M.TaqI to label the adenine and protect from cleavage when the CpG within its recognition site is methylated. Additionally, the assay shows over 90% labeling efficiency even at a 1:64 ratio of M.TaqI to labeling sites, emphasizing the catalytic performance of this DNA MTase enzyme (Figure S3A). This assay corroborated the imaging results (Figure S1B), showing that the labeling reaction is blocked by CpG methylation.

To verify that M.TaqI labels all non-methylated TCGA sites regardless of the local density of methylated CpG sites, we experimentally generated five samples of λ-DNA with increasing levels of CpG methylation using the CpG MTase M.SssI at various enzyme-per-site ratios. The increased amounts of methylation were verified using a methylation-sensitive restriction enzyme (R.BstUI), and a similar gel electrophoresis assay as described above (Figure S3C, left panel). Next, all samples were reacted with constant amounts of M.TaqI and AdoYnTAMRA, sufficient to drive the labeling reaction to completion. The degree of M.TaqI modification was verified using R.TaqI restriction enzyme, as described above. Examination of the fragment sizes after R.TaqI restriction, using gel electrophoresis, showed that higher CpG methylation results in greater digestion by R.TaqI, caused by a decrease in the number of sites that were available to M.TaqI modification. We also performed a control experiment in the absence of the AdoYnTAMRA cofactor. Complete fragmentation is observed for all samples, indicating that protection against fragmentation by R.TaqI is caused by transfer of the TAMRA fluorophore. In addition, we estimated CpG methylation and TAMRA labeling levels by comparing the experimental fragmentation patterns to simulated fragmentation patterns. Simulations were performed for R.BstUI at different CpG methylation levels and for R.TaqI at different adenine modification levels within the TCGA sequences (Supplementary file 2). The estimated TAMRA labeling levels are plotted against estimated CpG methylation levels in Figure S3C (right panel) and the obtained negative linear relationship indicates that TAMRA labeling directly depends on the CpG methylation. Single λ-DNA molecules with different CpG methylation/TAMRA labeling levels were also stretched on mixed hydrophilic/hydrophobic surface-modified cover-slips and analyzed by fluorescence microscopy (Figure S3D). The fluorescence images of individual λ-DNA molecules clearly show that the labeling density decreases with increasing CpG methylation. Taken together, these results strongly suggest that M.TaqI labeling is highly efficient at labeling non-methylated sites regardless of surrounding CpG methylation density.

Finally, we performed a similar protection assay to check whether the labeling reaction is also blocked by 5-hydroxymethylcytosine (5-hmC), an oxidized form of 5-methylcytosine (5-mC) which is also common in mammalian genomes. To this end, we amplified a 1052 bp segment from λ-DNA that contains only one TCGA site. Using PCR with either standard dCTP nucleotides or artificial d5-hmCTPs we generated two products with one non-modified/hydroxymethylated-modified TCGA site. We incubated both products with M.TaqI and AdoYnCF640 (a fluorescent analog of AdoYnTAMRA carrying the CF640 fluorophore). The labeling was verified by a restriction-protection assay (Figure S3B). Since the DNA segment in this experiment had only one M.TaqI/R.TaqI site, DNA that was not labeled was digested by R.TaqI into two fragments of 935 and 117 bp, the latter being very distinct in the gel assay. The results in Figure S3B show that when the cytosine in the TCGA site is hydroxymethylated, the DNA is not

protected from R.TaqI digestion (lane 3) while non-modified DNA is protected against cleavage by R.TaqI (lane 4). Hence, the hydoxymethylated DNA was not labeled by M.TaqI (the same behavior was observed for the control of modified DNA that was reacted only with R.TaqI, lane 5). These data indicate that M.TaqI labeling is sensitive to the 5-hmC modification, potentially allowing simultaneous 5-hmC and 5-mC mapping in mammalian DNA.

Reaction conditions for this assay: M.TaqI labeling (Figure S3B, samples 1-4) was performed in 30 µL NEB cutsmart buffer, 40 µM AdoYnCF640, 8.6 ng/µL M.TaqI, and 16.7 ng/µL PCR product. Samples 1, 3, and 5 contained 5-hmC-modified cytosines. Samples 5 and 6 were reacted in the same conditions without the presence of M.TaqI. After 1 h incubation at 60 °C, 0.5 µL R.TaqI was added to samples 3-6 and incubated for 1 h at 65 °C. Reactions were analyzed using agarose gel electrophoresis in the same manner as described above.

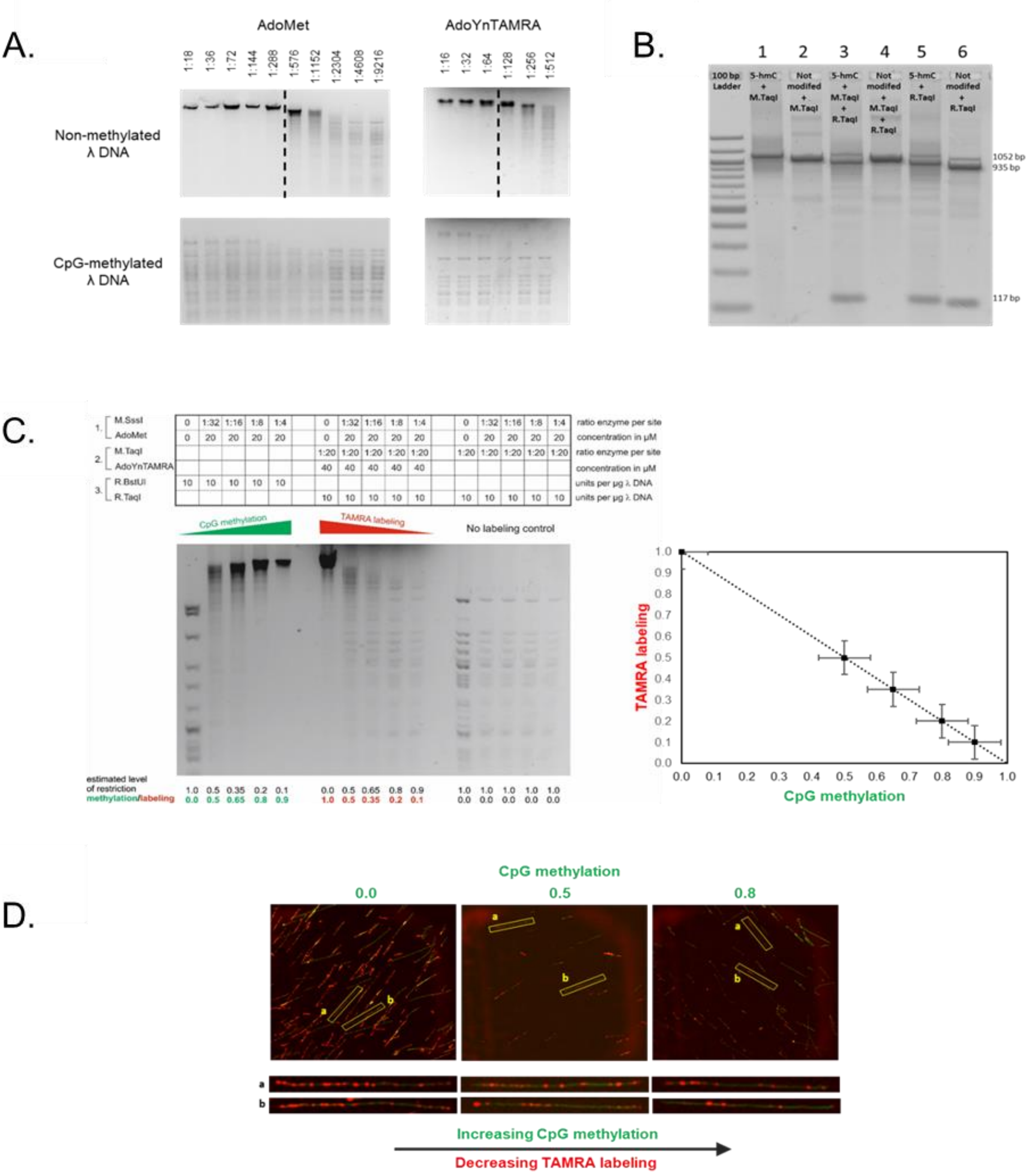
A.
AdoMet
AdoYnTAMRA
Non-methylated λ DNA
CpG-methylated λ DNA
B.
100 bp Ladder
5-hmC + M.TaqI
Not modifed + M.TaqI
5-hmC + M.TaqI + R.TaqI
Not modifed + M.TaqI + R.TaqI
5-hmC + R.TaqI
Not modifed + R.TaqI
1052 bp
935 bp
117 bp
C.
CpG methylation
TAMRA labeling
No labeling control
TAMRA labeling
CpG methylation
D.
CpG methylation
0.0
0.5
0.8
Increasing CpG methylation
Decreasing TAMRA labeling

Figure S3. A. Gel images from protection assays that were performed to confirm M.TaqI sensitivity to cytosine methylation. Either non-methylated or CpG-methylated λ-DNA was reacted in the presence of the natural cofactor AdoMet (left panel) or the synthetic fluorescent cofactor AdoYnTAMRA (right panel) using decreasing amounts of M.TaqI (enzyme-per-sites ratio indicated above the lanes). DNA was then incubated with the adenine methylation-sensitive restriction enzyme R.TaqI. Finally, the reaction products were size-separated using agarose gel electrophoresis. The critical enzyme-to-sites ratio from where fragmentation is observed is represented by a dashed line. **B.** Protection assays that were performed to confirm M.TaqI sensitivity to cytosine hydroxymethylation. Either hydroxymethylated (5-hmC) or not modified PCR products were incubated with the synthetic fluorescent cofactor AdoYnCF640 and M.TaqI, treated with R.TaqI and size-separated using agarose gel electrophoresis. CpG-hydroxymethylated DNA was not modified, and thus restriction is observed. Samples 1-2 served as negative control for the restriction reaction (no R.TaqI) and samples 5-6 served as positive control for the restriction reaction (no M.TaqI). **C.** Five samples of λ-DNA were gradually CpG-methylated using increasing amounts of M.SssI enzyme (ratios per site are indicated at the top of the chart). Next, to verify methylation, a fraction of each sample was reacted with constant amounts of the CpG methylation-sensitive restriction enzyme R.BstUI. Gel electrophoresis of the samples indicates decreasing fragmentation and hence increasing CpG methylation of the samples. Next, the CpG-methylated samples were incubated with constant amounts of the fluorescent cofactor AdoYnTAMRA and M.TaqI and reacted with the restriction enzyme R.TaqI. To verify that the modification was indeed the product of the specific effect of M.TaqI and AdoYnTAMARA, identical incubations were performed without the cofactor. Throughout the left panel, the ratios of enzymes-per-site and amounts of cofactor or restriction enzymes are indicated above the corresponding lane used for each sample. CpG methylation and TAMRA labeling levels were estimated by comparison of the experimental fragmentation patterns to simulated fragmentation patterns and plotted against each other (right panel). **D.** Fluorescence microscopy images of DNA from c. DNA was combed using mixed hydrophilic/hydrophobic surface-modified cover-slips. The green channel shows the unspecific DNA intercalator YOYO-1 (excitation: 488 nm) and the red channel shows the TAMRA labeling (excitation: 561 nm). The highest number of labels was obtained for non-methylated DNA (left panel) while the amount of labels decreases with increasing CpG methylation levels (middle to right panel).

ROM labeling is reproducible across biological and technical replicates

To evaluate the reproducibility of M.TaqI labeling on genomic DNA, we performed ROM on two biological replicates and two technical replicates of the GM12878 cell line. The correlation between replicates was then examined on a genome-wide scale. To this end, we divided the human genome into non-overlapping 10 kbp windows, and separately counted the number of ROM labels and number of aligned molecules in each window, for each one of the replicates (BEDTools intersect). Since GM12878 is a female cell line, all windows corresponding to Chromosome Y were discarded from this analysis. The ratio of labels to molecules was then calculated for each window, and the ratios obtained for each replicate were compared on a genome-wide scale.

Scatter plots depicting the correlation between the biological and technical replicates are presented in Figure S4. Pearson correlation coefficients calculated for each comparison are detailed in Supplementary Table 1. These results demonstrate that ROM labeling is highly reproducible across samples.

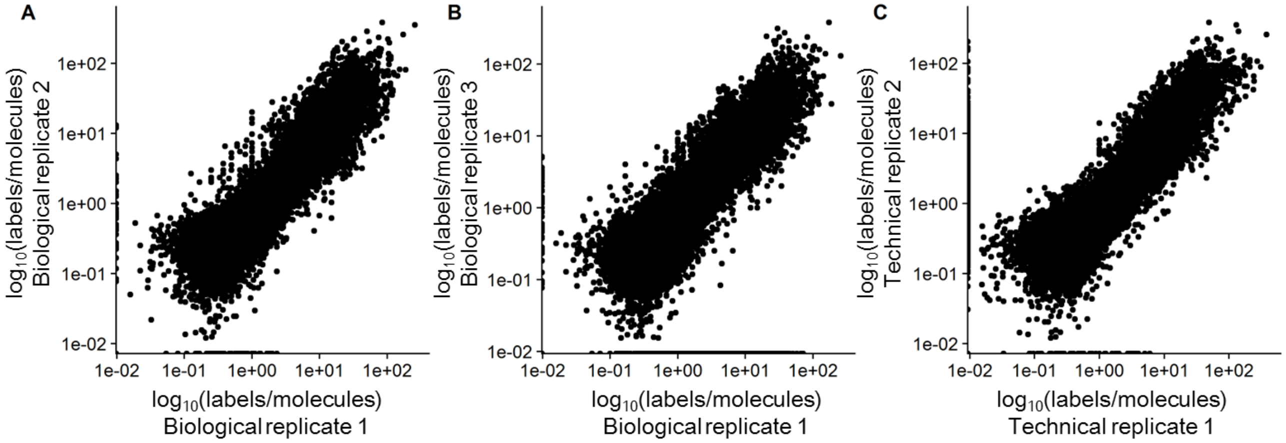

Figure S4. Scatter plots depicting the correlation of ROM labeling between biological and technical replicates of the GM12878 cell line. Each dot represents the ratio of detected labels to aligned molecules in a 10 kbp window. Axes are displayed in log_{10} scale. **A.** Comparison of biological replicate 1 and the first technical replicate of biological replicate 2. **B.** Comparison of biological replicate 1 and the second technical replicate of biological replicate 2. **C.** Comparison of technical replicates 1 and 2.

**Comparison**	**Pearson correlation coefficient**
Biological replicate 1, biological replicate 2 – technical replicate 1	0.738
Biological replicate 1, biological replicate 2 – technical replicate 2	0.693
Technical replicate 1, technical replicate 2	0.703

Table S1. Calculated Pearson correlation coefficients for comparison of ROM biological and technical replicates in 10 kbp windows across the human genome.

Genome-wide, locus-specific correlation between ROM and WGBS

To assess the genome-wide correlation between the ROM results and WGBS, we first divided the genome into non-overlapping windows of different sizes, ranging between 1.5 to 100 kbp (BEDTools makewindows), and compared the average WGBS signal in each window to the average ROM signal in the same window (calculated using BEDTools map). For each window size, we calculated Spearman's rank-order correlation values between the WGBS and ROM data in corresponding windows, including and excluding genomic windows with a missing non-methylation value in one of the methods. (Figure S5A). Correlation was relatively low for small bin sizes (1-10 kbp), probably due to the reduced-representation nature of ROM and its resolution limit, but increased for larger windows (50-100 kbp). The scatter plot depicting the correlation between the WGBS and ROM data in 100 kbp windows

(including zero-valued non-methylation signals) was colored according to the non-methylation level of the window in the WGBS data, to demonstrate the relationship between correlation in a bin and methylation content (Figure S5B). We denote that correlation was higher in windows with higher methylation content (low to medium non-methylation).

This approach to correlation analysis is limited by the fact that these two datasets are collected at different resolutions, and thus at different scales. To overcome this we used wavelet decomposition, a mathematical tool originating from signal processing, which in recent years has been applied to discover trends and correlations in continuous genomic data (see 'bioinformatics analysis pipelines' below).

A

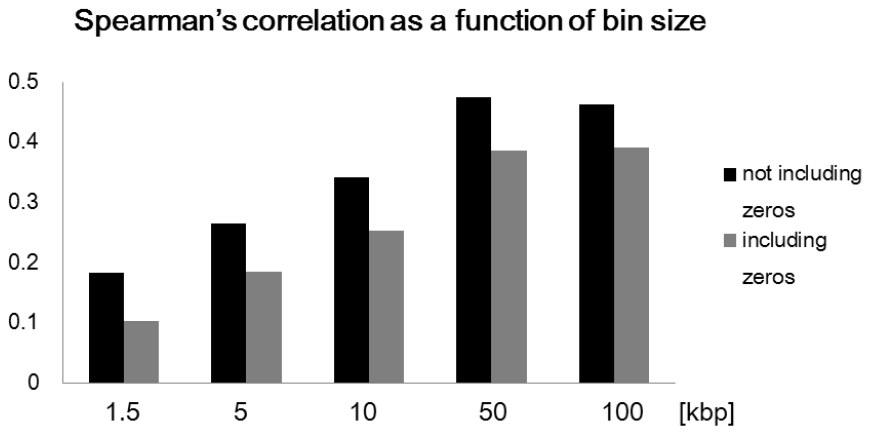

B

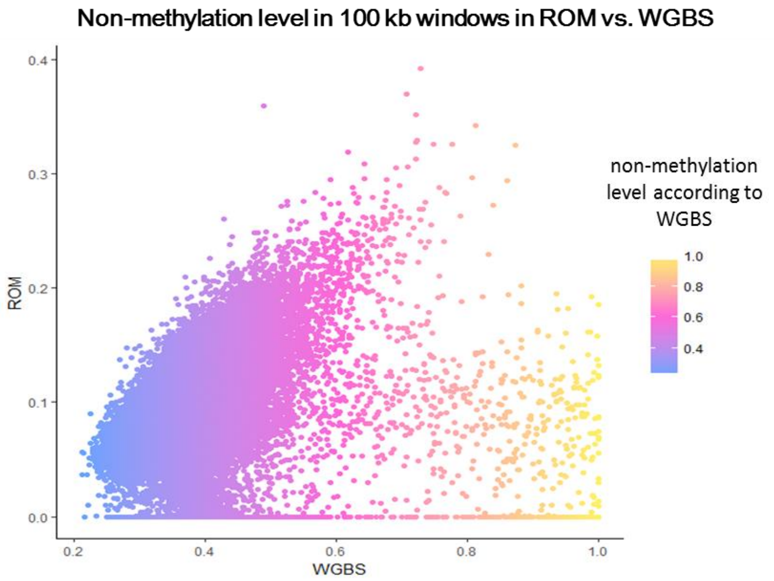

Figure S5. Genome-wide correlation between ROM and WGBS. **A.** Bar plot displaying Spearman's rank-order correlation values between the ROM and WGBS in varying window sizes. Black bars: correlation values not

including bin values equal to zero in one of the methods. Gray bars: correlation values including bin values equal to zero in one of the methods. **B.** Scatter plot of mean ROM and WGBS signal in non-overlapping 100 kbp windows along the human genome. Each dot represent one window. Dots are colored according to their methylation level in the WGBS data.

Second-generation sequencing, *de-novo* assembly, and read depth analysis

Purified BAC DNA was sheared using a Covaris AFA (Covaris Inc. Woburn, MA, USA), and the fragments were size-separated by electrophoresis on an agarose gel. Molecules within the range of 150-300 bp were extracted from the gel. DNA fragments were then adapted to Illumina sequencing using NEXTFlex kit (Bioo Scientific Corporation, Austin, TX, USA) and sequenced using a Miseq instrument (Illumina Inc., San Diego, CA, USA) to paired-end coverage of 15,000×. Sequencing reads were *de-novo* assembled using CLC Workbench software (CLC Bio-Qiagen, Aarhus, Denmark).

Since assembly of NGS reads did not yield any information about the repetitive region, read depth analysis was carried out for copy number quantification. In this type of analysis, the copy number of a certain repeat along the sequenced DNA is estimated based on the relative number of times it is represented in the sequencing data. This analysis is based on the assumption that all bases in the sequence are sequenced to the same extent. Thus, if a certain region is represented N times more than other regions in the sequencing read data, it follows that this region is also N times more abundant in the studied DNA.

Our analysis was based on the raw short reads obtained from sequencing. The contig produced by *de-novo* assembly was used as a reference sequence to which the reads were aligned.. This model contig represents the BAC sequence assuming a single repeat. Therefore, higher read depth along the repeat sequence compared to the non-repetitive sequence would indicate a greater number of repeats in the actual BAC. All reads were aligned to this reference sequence using Bowtie2 (Langmead and Salzberg 2012), and coverage was calculated using BEDTools (version 2.25.0) genomecov (Quinlan and Hall 2010). Coverage along the non-repetitive sequence was uniform, with an average of 14400±3255 reads per base (in-line with the calculated sequencing read depth) (Figure S6). However, coverage along the single repeat sequence was highly variable, averaging 139500±85500 reads per base (Figure S6). Averaged coverage values of both regions were compared to get a non-direct estimation of the number of repeats in the BAC. The ratio between coverage along the repetitive regions and the non-repetitive regions was approximately 8, implying that this is the number of repeats along the CH16-291A23 BAC (Figure 5b).

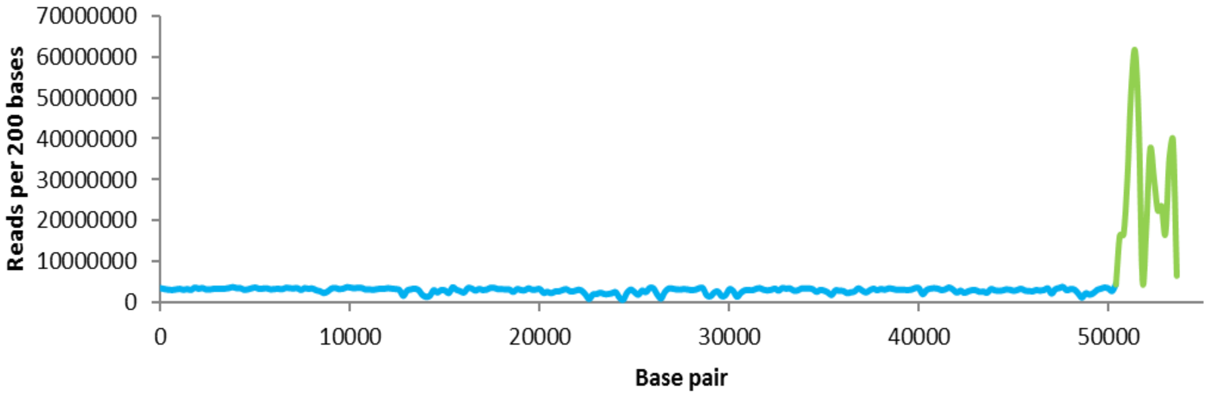

Figure S6. Read depth of sequencing reads aligned to the non-repetitive region of the BAC, containing the vector and the region upstream of the repeat region (light blue area), and one repeat (green area).

Methylation assessment of single repeat units along BAC DNA

For methylation analysis of repeat units along the CH16-291A23 BAC DNA, we performed ROM as an overlay on the repetitive genetic barcode. Figure S7A shows the unique pattern created by M.TaqI along the non-methylated BAC (Jeffet et al. 2016), highlighting the non-methylated repeat units. M.TaqI has two close-by recognition sites on each repeat unit that result in one visual label on each repeat, due to the optical diffraction limit. Nevertheless, when over-stretching the non-methylated BAC on modified microscope slides, the two green methylation labels flanking the red genetic label were clearly resolved, in agreement with the theoretical dual-color barcode (Figure S7B).

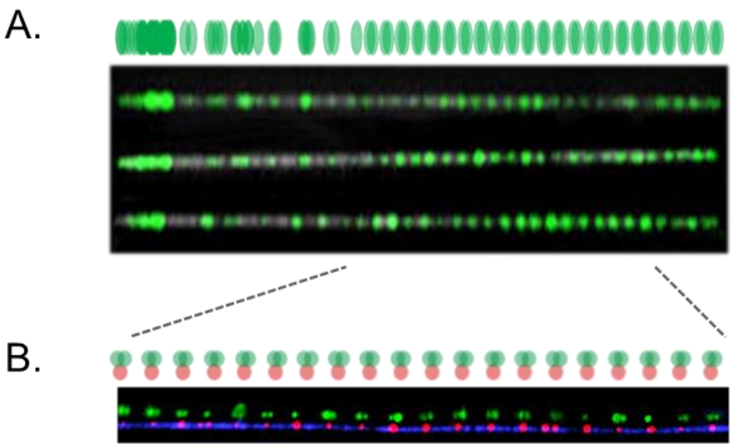

Figure S7. A. A reference map simulating the relative expected locations of ROM labels generated by M.TaqI along the stretched BAC (green). Below, images of three non-methylated, M.TaqI labeled BAC molecules linearized and stretched in nanochannels and aligned to the reference map. **B.** Partial genetic map (red) and ROM (green) from the repetitive region of the FSHD BAC stretched on a modified glass slide. The genetic identity and the number of repeat units are highlighted by labeling with the

nicking enzyme Nb.BsmI (red labels). Co-labeling the DNA with M.TaqI (green labels) indicated that the molecules were non-methylated. The displayed image is an overlay of red and green channels along the repetitive region of a single BAC molecule. Above the repeats is the reference map for the region. The green channel is shifted upwards to allow better visualization. The higher stretching factor achieved on modified glass compared to nanochannels enables detection of the two M.TaqI labels flanking the genetic label in each repeat.

DNA stretching and imaging on modified glass surfaces

Stretching of DNA in nanochannels, as facilitated by the Bionano Genomics technology, enables high-throughput, uniform stretching of chromosomal DNA molecules in an automated manner. The uniform stretching allows automated detection and analysis of the molecules as detailed in the methods section. However, these experiments are expensive and are only justified when high throughput and quantitative mapping is required. Thus, in some experiments DNA was stretched on glass slides for imaging. The main advantages of this technique are its simplicity and low cost and the fact that DNA is over-stretched (i.e., into lengths greater than its B-form contour length), improving resolution. In this report, two methods were used to facilitate DNA stretching on glass surfaces:

a. *Stretching on positively modified slides:* This method is based on the original optical mapping technique developed by Schwartz and coworkers (Cai et al. 1995). DNA stretching was performed on glass microscope cover slides that were pre-activated with an acid mixture and coated with positive and hydrophobic silanes, as previously reported (Michaeli et al. 2013), with minor modifications. Briefly, standard microscope cover slips were immersed overnight in a 2:1 (v/v) mixture of nitric acid (70%) and hydrochloric acid (37%) to facilitate cleaning and to break the Si-O bonds on the glass. Next, the slides were washed with ultrapure water, followed by ethanol (98%), and were dried with a nitrogen stream. Slides were then soaked in 300 mL of an aqueous solution containing 1,200 µL N-trimethoxysilylpropyl-N,N,N-trimethylammoniumchloride (Gelest, Inc., Morrisville, PA, USA) and 180 µL vinyltrimethoxysilane (Gelest) over night at 65 °C with mild shaking. This results in silane binding to hydroxyl groups on the glass surface. Slides were then washed with ultrapure water and ethanol (98%), and stored in ethanol (98%) at 4 °C. Before use, slides were washed with ultrapure water and dried. For stretching, the DNA sample solution (8 µL) was applied to the interface between an activated cover slip and a standard microscope slide and pulled into the space between the two surfaces by capillary forces. As DNA flows across the surface, the positive silanes serve as anchoring spots for the DNA, and the capillary flow unravels the DNA, resulting in immobilization of the molecules in a stretched form (Figure S1 & Figure 5C).

b. *Stretching on hydrophobic slides:* This method is based on the DNA molecular combing technique developed by Bensimon and coworkers (Herrick and Bensimon), but slides were rendered hydrophobic by coating them with the hydrophobic polymer Zeonex (Zeon, Tokyo, Japan), as described previously (Deen et al. 2015). First, 10 mM MES buffer (Sigma-Aldrich, Rehovot, Israel) was added to the DNA sample, resulting in a slightly acidic pH. Then, 10 µL of the DNA sample solution was incubated on the glass surface for 5 minutes to bind DNA tails to the surface. After this incubation, the drop was mechanically dragged on the surface, resulting in DNA stretching.

The DNA was either λ (Figure S1) or BAC DNA (Figure 5) at low concentration (~0.5 ng/µL). To visualize the DNA, each sample was stained with 0.5 µM of YOYO-1 (Invitrogen, Carlsbad, CA, USA), and 200 mM DTT (Sigma-Aldrich) were added to prevent photo damage.

Post-stretching, the DNA was imaged using an epifluorescence microscope (FEI Munich GmbH, Germany) equipped with a high-resolution EMCCD camera (IXon888, Andor Technology Ltd, Belfast, UK). A 150 W Xenon lamp was used for excitation (FEI Munich GmbH, Germany) with filter sets of 485/20ex and 525/30em, 560/25ex and 607/36em, and 650/13ex and 684/24em (Semrock Inc., Rochester NY, USA) for the YOYO-1, TAMRA, and Atto-647 channels, respectively.

Probability of detecting copy number information as a function of coverage

Optical mapping allows complex genomic information, such as large structural and copy number variations, to be read directly from individual, un-amplified, long fragments of DNA. Here, the number of repeats in the D4Z4 array in both alleles of an FSHD patient and a healthy control was counted (Figure 6). In order to reliably report on the number of repeats, at least one molecule spanning the complete investigated region needs to be sampled. Therefore, to estimate the required coverage for copy number analysis, we calculated the probability of sampling regions of varying lengths in a typical experiment, and simulated this probability for experiments with reduced coverage by randomly sub-sampling the data.

We estimated that a contracted D4Z4 array, containing at most 10 repeat units, would span approximately 50 kbp including the flanking regions needed for unambiguous alignment. We therefore

divided the genome (build hg19) into non-overlapping 50 kbp windows (BEDTools makewindows), and calculated the percentage of 50 kbp regions completely covered by at least one molecule in a typical experiment where 87× coverage was generated (BEDTools intersect). Since this calculation was performed on data obtained for the female cell line GM12878, Chromosome Y was excluded from this analysis. This calculation was performed once genome-wide, and a second time excluding windows containing N-base gaps of 10 kbp or longer in the reference, which hinder molecule alignment (A list of N-base gaps is attached as Supplemental file 3). 93% of genome-wide 50 kbp windows were fully covered by at least one molecule, and percentages increased to 98% of regions when excluding N-base gaps. To evaluate how this percentages change as a function of coverage, molecules were randomly sub-sampled using an in-house script. Random sub-sampling was performed several times to simulate varying molecule coverage, using the following percentages of molecules: 10, 25, 50, and 75. Simulation for each percentage was repeated three times. This analysis showed that 91% of 50 kbp regions genome-wide, and 96% of 50 kbp regions excluding N-base gaps, were covered completely by at least one molecules at 20× coverage, much lower than the coverage obtained in a single typical experiment (Figure S8).

To investigate the correlation between the length of the investigated region and the probability to sample at least one molecule spanning the region in its entirety, a similar simulation was performed using windows of varying lengths sampled from Chromosomes 4 and 10. For this simulation, an aggregated dataset of three typical experiments with 231× average coverage was used, and 5000 regions across each chromosome were sampled randomly. The probability of capturing at least one molecule that completely spans the interval of interest goes down with interval length (Table S2). This is expected, as the probability of sampling molecules of increasing lengths goes down exponentially.

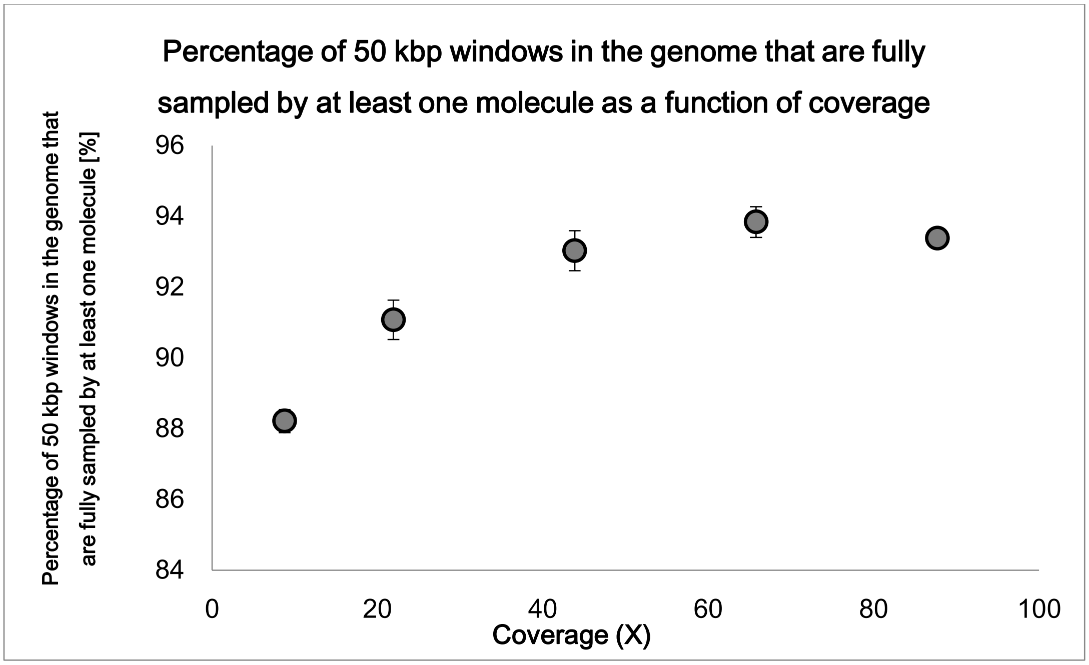

Figure S8. Graph displaying the percentage of 50 kbp genomic windows that were completely covered by at least one molecule in a typical experiment, and in randomly sub-sampled datasets of molecules from the same experiment resulting in decreased coverage.

	Windows length			
	10 kbp	50 kbp	100 kbp	200 kbp
Chromosome 4	99.7%	99.4%	97.4%	88.4%
Chromosome 10	97.2%	96.8%	95.1%	85.6%

Table S2. Probabilities of sampling at least one molecule that spans the entire region, for different region lengths, in two chromosomes in an aggregated dataset of three typical experiments with an average coverage of 231×.

ROM analysis of patient previously diagnosed with FSHD

To demonstrate the diagnostic potential of ROM, we performed ROM on DNA extracted from whole blood of a patient previously diagnosed with FSHD. *De-novo* assembly of single molecules resulted in two contigs, allowing us to distinguish the 4qA and 4qB alleles (Figure S9), each containing a distinct copy number of D4Z4 repeats. The detected ROM methylation-sensitive labels indicated that both alleles were non-methylated. Moreover, based on the equally-spaced ROM labels, we were able to detect five repeats on the 4qA allele, and eight on the non-pathogenic 4qB allele (Lemmers et al. 2007).

Previous genetic testing based on pulsed-field gel electrophoresis estimated approximately four repeats for the 4qA allele, and approximately seven repeats for the 4qB allele. To resolve this discrepancy, we measured the length in kbp corresponding to the entire macrosatellite array in the optical consensus maps of the two alleles, and found that the arrays are shorter than expected from intact tandem repeats. This variation is most likely due to partial truncation of the first or last repeat in the array, as commonly exhibited in repeat arrays (Satovi et al. 2016).

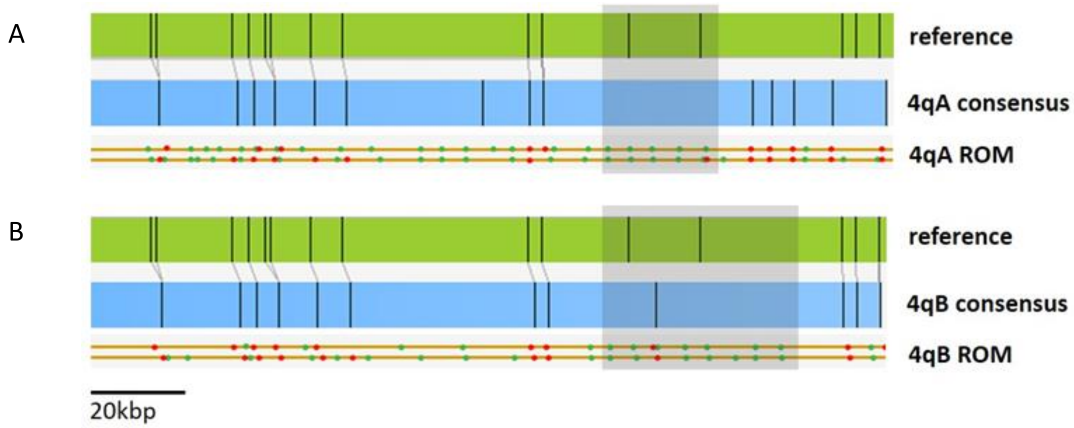

Figure S9. Copy number and methylation analysis of the pathogenic contraction on chromosome arm 4q of an FSHD patient, enabled by *de-novo* assembly of single DNA molecules extracted from a whole-blood sample. Digitized representation of single molecules is shown as yellow lines, with detected genetic labels as red dots and ROM non-methylation labels as green dots. The two resulting assembled contigs are shown as blue horizontal bars, with consensus genetic label positions shown as black vertical lines. The *in-silico* generated reference map of the investigated region is shown as a green horizontal bar, with calculated expected nicking sites as black vertical lines. Alignment between the assembled contigs and the reference is indicated by grey lines between the blue and green bars. The expected position of the D4Z4 array is indicated by a grey box **A.** Consensus map corresponding to the 4qA allele. Each green dot inside the gray box corresponds to one repeat, indicating five repeat units. **B.** Consensus map corresponding to the 4qB allele. Each green dot inside the gray box corresponds to one repeat, indicating eight repeat units.

ROM and RRBS

ROM offers reduced methylation representation, reporting on the methylation state of CpGs nested within TCGA sites in the genome. This is conceptually similar to RRBS, where CCGG sites are cleaved and size-selected fragments of ~10-200 bp are collected and sequenced. This results in bp resolution regarding CpG methylation, but only for these enriched regions. While in ROM all TCGA sites are assessed, in RRBS methylation of CpGs is measured between two cleaved CCGG sites. Moreover, for RRBS it is ideal that CCGG sites are dense, since short fragments are enriched. For ROM, on the other

hand, sites should be about one kbp apart to offer good optical resolution. Hence, the coverage provided in both methods cannot be compared directly by looking at the number of CCGG and TCGA sites in various regions (Table S3). However, the distances between neighboring sites can provide information regarding parts of the genome represented by each method. Pairwise distances between sites in promoters, gene bodies, and CGIs were calculated for both TCGA and CCGG sites and are presented as histograms in Figure S10. Although both sites contain four nucleotides, their distribution within the examined regions is extremely different: while CCGG sites seem to be clustered, TCGA sites appear at greater distances from each other, making them more suitable for optical measurements. The different distribution of information captured by the two techniques makes them complementary, and choice of usage depends on the desired information. Both ROM and RRBS can be performed with other enzymes that probe different recognition sites and thus different genomic information.

	Promoters	CGIs	Genes
% of TCGA sites of all CpGs	3.4	1.5	3.4
% of CCGG sites of all CpGs	11.3	13.2	11.3

Table S3. The percentage of TCGA and CCGG sites out of total CpG sites (both TCGA and CCGG sites contain a CpG site at their second and third bases) calculated for promoters, gene bodies and CGIs. The percentage was calculated for every single element and then averaged over all the elements in the genome.

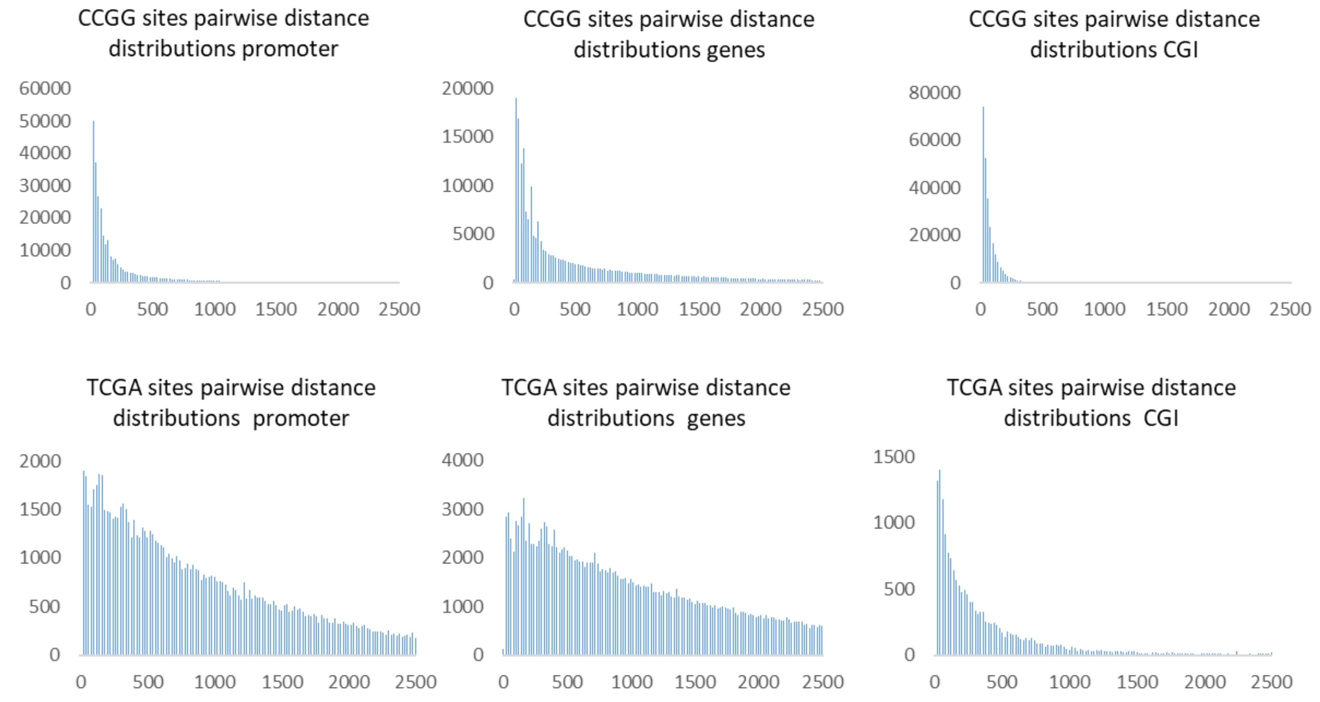

Figure S10. Histograms of distances between either CCGG sites (top) or TCGA sites (bottom) as calculated for promoters (left charts), gene bodies (middle charts) and CpG islands (right charts). The X-axis represents the pairwise distance between sites in bp and the Y-axis the number of distances accurances.

Bioinformatics analysis pipelines

To generate ROM profiles along the human genome and the BAC sequence, all molecules were first aligned to the corresponding reference sequence, based on their genetic map. Alignment was performed using Irys view (Bionano Genomics, Inc., San Diego, California, USA). Methylation maps were created by aligning each molecular methylation profile to the genomic locus defined by the genetic map on the same molecule. To account for optical resolution, each M.TaqI label position was extended to a size of 1 kbp using BEDTools slop. We then counted the total number of labels at each genomic position, as well as the total number of molecules aligned to each position using BEDTools genomecov. A methylation score for each genomic position was calculated by dividing the total number of labels in a given bp by the total number of aligned molecules (simply, a score of 1 would mean that all the molecules covering a certain locus were labeled, while 0 would mean that none of the molecules had such a label).

WGBS data for primary peripheral blood mononuclear cells (PBMC) was obtained from the “MethBase” methylome database by the Smith lab (Song et al. 2013). For visual assessment of the profile similarities

between ROM and WGBS data, CpG methylation scores from the WGBS data were converted to non-methylation scores, reflecting the level of non-methylated CpGs in a given position.

Methylation profiles over genes and regulatory histone marks were generated as follows: Gene locations were defined according to RefSeq annotation for hg19, downloaded from the UCSC Genome Browser database. H3K4me3 and H3K9Ac ChIP-seq data for PBMCs was obtained from the "epigenome road map project" (GEO accession numbers GSM1127127 and GSM613883, respectively), and converted into genomic coverage using BEDTools genomecov.
To classify genes by their methylation level (Figure 2B), the average methylation, as detected by WGBS, was calculated for each gene using BEDTools map. High methylation in genes was defined as normalized WGBS non-methylation values of 0-0.3, medium methylation as 0.3-0.5, and low methylation as 0.5-1. Mean non-methylation values along genes and around histone modifications were calculated for the inverted WGBS data and the normalized ROM methylation scores (as described above) using deepTools (Ramírez et al. 2016) computeMatrix. Non-methylation along genes was calculated in scale regions mode, where each gene was scaled to 15 kbp and divided into 300 bp bins. For histone modifications calculation was performed in reference-point mode, where each histone modification midpoint was extended 3 kbp up- and downstream, and the region was divided into 50 bp bins. In both cases, the mean non-methylation score in each bin was calculated, and all scores in the same bin were summed. The score for gene bodies was normalized to the number of genes in each group. In both cases, normalization of values to a 0-1 range was performed separately for the ROM and WGBS datasets.

For genome-wide correlation assessments between WGBS and ROM data, we divided the genome into non-overlapping windows of varying lengths. As WGBS data contains information solely on CpG dinucleotides, we assigned each CpG in the genome a ROM value, based on the genome-wide ROM profile that was generated as described above. Then, for each window size, mean ROM and WGBS non-methylation levels were calculated in each window using BEDTools map. Thus, the score in each genomic window is normalized to the number of CpGs in that window.

For wavelet decomposition, we first used the discrete wavelet transform to bring the WGBS data to a more coarse scale, similar to that of the ROM data. Then, the continuous wavelet transform was used to decompose both datasets into a series of coefficients. The correlation at each genomic position was then calculated as the Pearson correlation between these wavelet coefficients in a 10 kb window centered at that position. For wavelet analysis, the WGBS data was smoothed using the MODWT function in the R package waveslim, using the la8 wave function and reflection boundary conditions.

Wavelet decomposition was performed using the first derivative of a Gaussian (DOG) function in the R package Rwave. Pearson correlation between wavelet decomposition coefficients was performed using the SciPy implementation of this analysis.

For analysis of regions with a high wavelet decomposition correlation score, we focused on all regions with a correlation score equal or exceeding 0.5. For each such identified region, we calculated its midpoint and added 5 kbp upstream and downstream of that point (BEDTools slop). Overlapping regions were than merged using BEDTools merge. The mean non-methylation scores for both WGBS and ROM data in these regions was calculated using BEDTools map, and Spearman's rank-order correlation values were calculated.

Examination of represented functional elements in high correlation regions was performed using BEDTools intersect. Transcription start sites (TSS) and gene bodies were defined according to RefSeq annotation for hg19 downloaded from the UCSC Genome Browser database. Positions of CpG islands were also downloaded from the UCSC Genome Browser. Positions of TCGA sites along the genome were generated using the BSgenome R package. Promoters were defined as ±2 kbp from TSS. The same approach was used to calculate the percentage of M.TaqI sites (TCGA sites) in gene promoters. We note that since available WGBS data is aligned to build hg19, optical data was aligned to the same reference for comparison.

Long-range methylation profiles of single molecules were visualized on Integrative Genomics Viewer (IGV), against the reference genome build hg19. Density of M.TaqI sites (TCGA sites) in a 1.5 kbp sliding window was generated using an in-house script.

Average coverage of a ROM experiment was calculated by dividing the total lengths of molecules that aligned to the reference by the total length of the genome.

REFERENCES

Cai W, Aburatani H, Stanton VP, Housman DE, Wang YK, Schwartz DC. 1995. Ordered restriction endonuclease maps of yeast artificial chromosomes created by optical mapping on surfaces. *Proc Natl Acad Sci U S A* **92**: 5164–8.

Deen J, Sempels W, De Dier R, Vermant J, Dedecker P, Hofkens J, Neely RK. 2015. Combing of genomic DNA from droplets containing picograms of material. *ACS Nano* **9**: 809–16.

Grunwald A, Dahan M, Giesbertz A, Nilsson A, Nyberg LK, Weinhold E, Ambjörnsson T, Westerlund F, Ebenstein Y. 2015. Bacteriophage strain typing by rapid single molecule analysis. *Nucleic Acids Res* **43**: e117–e117.

Hansen KD, Sabunciyan S, Langmead B, Nagy N, Curley R, Klein G, Klein E, Salamon D, Feinberg AP. 2014. Large-scale hypomethylated blocks associated with Epstein-Barr virus – induced B-cell immortalization. 177–184.

Hanz GM, Jung B, Giesbertz A, Juhasz M, Weinhold E. 2014. Sequence-specific Labeling of Nucleic Acids and Proteins with Methyltransferases and Cofactor Analogues. *J Vis Exp* 3–12.

Herrick J, Bensimon A. Chapter 5 Introduction to Molecular Combing : Genomics , DNA Replication , and Cancer. **521**.

Jeffet J, Kobo A, Su T, Grunwald A, Green O, Nilsson AN, Eisenberg E, Ambjörnsson T, Westerlund F, Weinhold E, et al. 2016. Super-Resolution Genome Mapping in Silicon Nanochannels. *ACS Nano* **10**: 9823–9830.

Klimasauskas S, Weinhold E. 2007. A new tool for biotechnology: AdoMet-dependent methyltransferases. *Trends Biotechnol* **25**: 99–104.

Langmead B, Salzberg SL. 2012. Fast gapped-read alignment with Bowtie 2. *Nat Methods* **9**: 357–9. http://www.ncbi.nlm.nih.gov/pubmed/22388286%0Ahttp://www.pubmedcentral.nih.gov/articlerender.fcgi?artid=PMC3322381.

Lemmers RJLF, Wohlgemuth M, van der Gaag KJ, van der Vliet PJ, van Teijlingen CMM, de Knijff P, Padberg GW, Frants RR, van der Maarel SM. 2007. Specific Sequence Variations within the 4q35 Region Are Associated with Facioscapulohumeral Muscular Dystrophy. *Am J Hum Genet* **81**: 884–894. http://linkinghub.elsevier.com/retrieve/pii/S000292970763866X.

McClelland M, Nelson M. 1992. Effect of site-specific méthylation on dna modification methyltransferases and restriction endonucleases. *Nucleic Acids Res* **20**: 2145–2157.

Michaeli Y, Shahal T, Torchinsky D, Grunwald A, Hoch R, Ebenstein Y. 2013. Optical detection of epigenetic marks: sensitive quantification and direct imaging of individual hydroxymethylcytosine bases. *Chem Commun* **49**: 8599. http://xlink.rsc.org/?DOI=c3cc42543f.

Quinlan AR, Hall IM. 2010. BEDTools: a flexible suite of utilities for comparing genomic features. *Bioinformatics* **26**: 841–2.

Ramírez F, Ryan DP, Grüning B, Bhardwaj V, Kilpert F, Richter AS, Heyne S, Dündar F, Manke T. 2016. deepTools2: a next generation web server for deep-sequencing data analysis. *Nucleic Acids Res* **44**: W160–W165. https://academic.oup.com/nar/article-lookup/doi/10.1093/nar/gkw257.

Satovi E, Zeljko TV, Luchetti A, Mantovani B, Plohl M. 2016. Adjacent sequences disclose potential for intra-genomic dispersal of satellite DNA repeats and suggest a complex network with transposable elements. 1–12.

Song Q, Decato B, Hong EE, Zhou M, Fang F, Qu J, Garvin T, Kessler M, Zhou J, Smith AD. 2013. A Reference Methylome Database and Analysis Pipeline to Facilitate Integrative and Comparative Epigenomics. **8**.
