# Supplementary material for: Long-read single-molecule maps of the functional methylome
Source: Genome Res. 2019 Apr;29(4):646–56. doi: 10.1101/gr.240739.118 (PMC6442387; doi:10.1101/gr.240739.118)
Supplement: Supplemental Material [file supp_gr.240739.118_Supplemental_code_CWT_DOG_calc.R.html]

Supplemental\_code\_CWT\_DOG\_calc 

# Long-read single-molecule maps of the functional methylome
