# Supplementary material for: Long-read single-molecule maps of the functional methylome
Source: Genome Res. 2019 Apr;29(4):646–56. doi: 10.1101/gr.240739.118 (PMC6442387; doi:10.1101/gr.240739.118)
Supplement: Supplemental Material [file supp_gr.240739.118_Supplemental_File_4.docx]

The following are the R scripts used:

1. **methometeR_Location_Calculation_Positive_Strand.r and methometeR_Location_Calculation_Negative_Strand.**r : Takes as input the human reference cmap, Molecule query cmap and Molecule xmap, to estimate the chromosomal position of the labels pertaining to methylation (Label Channel=1). The chromosomal position of the nick sites (Label Channel=2), is calculated by first getting the molecule id from the molecule cmap, getting the site ids of nick site of the molecule corresponding to the reference genome using the contig xmap and finally extracting the chromosomal locations from the human reference cmap, using the site id. Methylation locations on the chromosome are calculated using the chromosome location of the nick site and the distance between methylation and nick site on the contig. Output is a modified Molecule query cmap data containing the 3 additional columns; NickLoc and MethLoc which keeps a count of nick and methylation labels and chromosome position which contains the nick and methylation chromosomal position.
2. **MethylationCount_and_Normalization.r** : Input is the modified contig query file, number of repeats and repeat size. Calculates the number of molecules spanning each repeat region (size 3.3 Kilo Bases) and finds the number of methylation label (Label Channel=1) in each repeat regions. Then normalizes the methylation counts, by dividing number of methylation labels in each repeat region with the number of molecules spanning that repeat. Output is a file containing the chromosomal region, the repeat number, the number of methylation labels, number of molecules spanning each repeat, and the normalized counts.
