# Supplementary material for: Long-read single-molecule maps of the functional methylome
Source: Genome Res. 2019 Apr;29(4):646–56. doi: 10.1101/gr.240739.118 (PMC6442387; doi:10.1101/gr.240739.118)

Estimation of modification efficiency by comparison to simulated fragmentation profiles

1.	M.SssI	0	1:32	1:16	1:8	1:4	0	1:32	1:16	1:8	1:4	ratio enzyme per site
	AdoMet	0	20	20	20	20	0	20	20	20	20	concentration in µM
2.	M.TaqI						1:20	1:20	1:20	1:20	1:20	ratio enzyme per site
	AdoYnTAMRA						40	40	40	40	40	concentration in µM
3.	R.BstUI	10	10	10	10	10						units per µg λ DNA
	R.TaqI						10	10	10	10	10	units per µg λ DNA

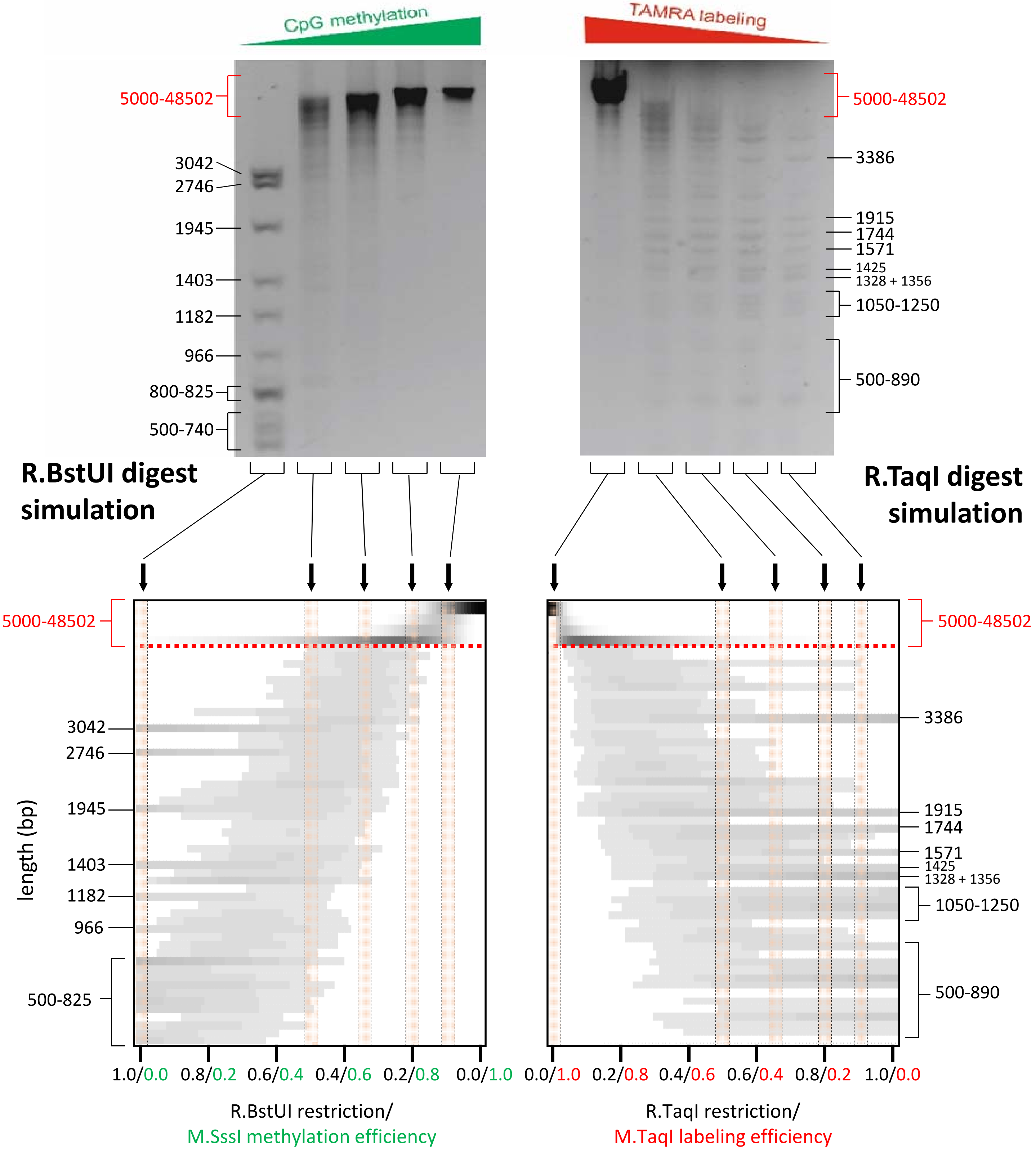

Supplement: Supplemental Material [file supp_gr.240739.118_Supplemental_File_2.pdf]
